# Combination of polymeric micelle formulation of TGFβ receptor inhibitors and paclitaxel produces consistent response across different mouse models of Triple‐negative breast cancer

**DOI:** 10.1002/btm2.10681

**Published:** 2024-06-04

**Authors:** Natasha Vinod, Duhyeong Hwang, Sloane Christian Fussell, Tyler Cannon Owens, Olaoluwa Christopher Tofade, Thad S. Benefield, Sage Copling, Jacob D. Ramsey, Patrick D. Rädler, Hannah M. Atkins, Eric E. Livingston, J. Ashley Ezzell, Marina Sokolsky‐Papkov, Hong Yuan, Charles M. Perou, Alexander V. Kabanov

**Affiliations:** ^1^ Division of Pharmacoengineering and Molecular Pharmaceutics, Center for Nanotechnology in Drug Delivery, Eshelman School of Pharmacy University of North Carolina at Chapel Hill Chapel Hill North Carolina USA; ^2^ Joint UNC/NC State Department of Biomedical Engineering University of North Carolina Chapel Hill North Carolina USA; ^3^ College of Pharmacy Keimyung University Daegu Republic of Korea; ^4^ Department of Biology, Department of Chemistry University of North Carolina at Chapel Hill Chapel Hill North Carolina USA; ^5^ Department of Radiology University of North Carolina at Chapel Hill Chapel Hill North Carolina USA; ^6^ Lineberger Comprehensive Cancer Center University of North Carolina Chapel Hill North Carolina USA; ^7^ Department of Genetics University of North Carolina Chapel Hill North Carolina USA; ^8^ Pathology and Laboratory Medicine, School of Medicine University of North Carolina Chapel Hill North Carolina USA; ^9^ Department of Pathology and Laboratory Medicine, Division of Comparative Medicine University of North Carolina Chapel Hill North Carolina USA; ^10^ Center for Human Health and the Environment North Carolina State University Raleigh North Carolina USA; ^11^ Department of Radiology, Biomedical Research Imaging Center, UNC Lineberger Comprehensive Cancer Center University of North Carolina at Chapel Hill Chapel Hill North Carolina USA; ^12^ Histology Research Core University of North Carolina Chapel Hill North Carolina USA; ^13^ Present address: National Cancer Institute, National Institutes of Health Bethesda Maryland USA; ^14^ Present address: Vaccine Research Center, National Institute of Allergy and Infectious Disease National Institutes of Health Bethesda Maryland USA

## Abstract

Triple‐negative breast cancer (TNBC) is notoriously difficult to treat due to the lack of targetable receptors and sometimes poor response to chemotherapy. The transforming growth factor beta (TGFβ) family of proteins and their receptors (TGFRs) are highly expressed in TNBC and implicated in chemotherapy‐induced cancer stemness. Here, we evaluated combination treatments using experimental TGFR inhibitors (TGFβi), SB525334 (SB), and LY2109761 (LY) with paclitaxel (PTX) chemotherapy. These TGFβi target TGFR‐I (SB) or both TGFR‐I and TGFR‐II (LY). Due to the poor water solubility of these drugs, we incorporated each of them in poly(2‐oxazoline) (POx) high‐capacity polymeric micelles (SB‐POx and LY‐POx). We assessed their anticancer effect as single agents and in combination with micellar PTX (PTX‐POx) using multiple immunocompetent TNBC mouse models that mimic human subtypes (4T1, T11‐Apobec and T11‐UV). While either TGFβi or PTX showed a differential effect in each model as single agents, the combinations were consistently effective against all three models. Genetic profiling of the tumors revealed differences in the expression levels of genes associated with TGFβ, epithelial to mesenchymal transition (EMT), TLR‐4, and Bcl2 signaling, alluding to the susceptibility to specific gene signatures to the treatment. Taken together, our study suggests that TGFβi and PTX combination therapy using high‐capacity POx micelle delivery provides a robust antitumor response in multiple TNBC subtype mouse models.

**Translational Impact Statement**

Paclitaxel (PTX) is a widely used chemotherapy in breast cancer. However, response to single‐agent chemotherapy is short‐lived in a metastatic setting. This study shows the broad applicability of the therapeutic combination of transforming growth factor beta inhibitors with PTX across different triple‐negative breast cancer subtypes.

## 1 INTRODUCTION

According to the Global Cancer Statistics, there were 19 million new cancer cases and 10 million cancer deaths estimated worldwide in 2020.^1^ Female breast cancer accounted for 11.7% of all cancer types (2.3 million cases), making it the most common type of cancer in 2020 and the leading cause of cancer death in women. Triple‐negative breast cancers (TNBCs), characterized by the lack (<1%) of receptors for estrogen, progesterone, and lack of human epidermal growth factor 2 (HER‐2) overexpression, have the worst prognosis of all breast cancers. The analysis of gene expression patterns in different tumors has uncovered several main subtypes of breast cancer. These include basal‐like, luminal A, luminal B, HER‐2 enriched, and Claudin‐low.^2^ Unlike the luminal subtype, the basal‐like subtype is associated with poor prognosis and is distinguished from luminal tumors based on the expression of cytokeratins. The majority of TNBCs have a basal epithelial‐like phenotype and stain positive for cytokeratins 5/6 and 17.^3^ The Claudin‐low TNBC subtype, discovered in 2007 by Herschkowitz et al., is classified by the low expression of Claudin 3, 4, and 7 and other tight junction proteins such as E‐cadherin.^2^ In addition to the subtypes mentioned above, TNBC has been further divided into seven clusters on the basis of biological pathway activity, including immune signaling cascades and metabolic pathways.^4^

Due to the lack of targetable receptors and sometimes poor response to immunotherapy using immune checkpoint inhibitors (ICI), TNBC has been notoriously difficult to treat. Until recently, cytotoxic chemotherapy has been the mainstay of TNBC treatment.^5^ However, although TNBC responds to chemotherapy to some extent, the median overall survival with single‐agent chemotherapy is only 17.5 months in the metastatic setting.^6^ More recently developed targeted therapeutics, such as the poly(ADP‐ribose) polymerase inhibitors, Olaparib and Talazoparib, have shown promising outcomes and were approved in patients with BRCA mutations.^7^ However, around 40%–70% of the patients are likely to develop resistance to these therapies.^8^ Moreover, the significant level of heterogeneity of TNBC makes it practically challenging to stratify patients based on specific genomic and molecular signatures and identify candidates for targeted therapies. Besides, the heterogeneity of TNBC grows over time, resulting in the emergence of drug‐resistant cancer stem‐like cells.^9^ The high relapse rates and poor prognosis of TNBC have fueled efforts to find new treatments that could be effective across different TNBC subtypes.

Combining chemotherapy with ICI has shown promise for treating TNBC. Clinical trial results showed that pembrolizumab (anti‐PD1) had an improved response rate when paired with chemotherapy (nab‐paclitaxel or paclitaxel [PTX], or gemcitabine plus carboplatin) than when used alone. These results granted accelerated Food and Drug Administration (FDA) approval of pembrolizumab and chemo‐immunotherapy combinations for PDL1‐positive TNBCs.^10^ Atezolizumab (anti‐PDL1) in combination with nab‐paclitaxel also received an accelerated FDA approval for the treatment of patients with unresectable locally advanced or metastatic PDL1‐positive TNBC based on the initial Phase 3 clinical trial^11^; however, data from the subsequent additional Phase 3 trial showed that the combination of atezolizumab and PTX did not improve progression‐free survival compared to PTX alone (https://clinicaltrials.gov/ct2/show/NCT03125902). Subsequently, the combination of atezolizumab and chemotherapy was withdrawn (https://www.gene.com/media/press-releases/14927/2021-08-27/genentech-provides-update-on-tecentriq-u). Given this development, as well as the fact that ICI treatment is associated with severe immune‐related adverse events, the need for newer therapeutic strategies that can blend well with chemotherapy while causing fewer side effects remains important.

The transforming growth factor beta (TGFβ) is a superfamily of pleiotropic cytokines that regulate various biological processes, including cell growth, differentiation, immune regulation, and apoptosis.^12^ TGFβ signaling negatively regulates the inflammatory response of immune cells, thereby promoting tumor growth.^13^ Cancer cells exploit TGFβ signaling to promote EMT, immune tolerance, and myofibroblast differentiation.^14^ TGFβ proteins have recently been found to play a key role in regulating stemness in breast cancer cells, suggesting that it could be a crucial target in TNBC.^15^ Biopsies from breast cancer patients who received chemotherapy showed an increase in TGFβ and cancer stem cell (CSC) markers compared to biopsies obtained before the treatment.^16^ The expression of CSC gene signatures (CD44 and ALDH1A1) in human breast cancer cells with high autocrine TGFβ signaling can be downregulated by a TGFβ inhibitor (TGFβi) that targets TGFβ receptor‐I (TGFR‐I).^16^ These findings support the rationale for combining chemotherapy with inhibitors of TGFβ receptors in TNBC.

Here, we evaluated two small‐molecule TGFβi, SB525334 (SB) and LY2109761 (LY), that target TGFR‐I or both TGFR‐I and TGFβ receptor‐II (TGFR‐II), respectively. Since these drugs are water‐insoluble, we incorporated them in the polymeric micelles (PMs) of poly(2‐oxazoline) (POx) block copolymer to enable parenteral delivery of poorly soluble agents.^17^ We used the micellar drugs (SB‐POx and LY‐POx, respectively) as single treatments and in combination with PTX, which was also incorporated in POx PMs (PTX‐POx). For the therapeutic assessment, we employed syngeneic TNBC models that are representative of a hybrid basal‐like and Claudin‐low phenotype (4T1) and Claudin‐low phenotype (T11‐Apobec and T11‐UV).

## 2 MATERIALS AND METHODS

### 2.1 Materials

The triblock copolymer P[MeOx_36_‐*b*‐BuOx_21_‐*b*‐MeOx_35_]‐piperidine (POx) (*M*_w_/*M*_n_ [weight‐average molecular weight/number‐average molecular weight] = 1.04) was synthesized by ring‐opening polymerization of 2‐oxazolines as described previously.^18^ The block lengths were determined using ^1^H nuclear magnetic resonance (NMR) spectroscopy on a Bruker Avance III 400‐MHz spectrometer, and the NMR spectrum was analyzed using the MestReNova software (11.0). The molecular weight distribution of POx was obtained by gel permeation chromatography on a Viscotek VE2001 solvent sampling module. TGFβi, LY, and SB were purchased from Adooq BioScience and APExBIO, respectively. PTX was purchased from LC Laboratories. The remaining reagents were purchased from Sigma‐Aldrich or Fisher Scientific unless specified otherwise.

### 2.2 Cell culture

NIH‐3T3 mouse embryonic fibroblasts and 4T1 mammary carcinoma cell lines were obtained from the Tissue Culture Facility at UNC Lineberger Comprehensive Cancer Center (LCCC). The NIH‐3T3 cells were cultured in  Dulbecco's Modified Eagle Medium (Gibco) supplemented with 10% fetal calf serum and 1% penicillin and streptomycin at 37°C in a humidified atmosphere containing 5% CO_2_. The 4T1 TNBC cells were obtained from UNC LCCC Tissue Culture Facility and were cultured in the same way, except that the basal media was RPMI (Gibco), and fetal bovine serum (FBS) was used instead of fetal calf serum. The T11‐Apobec and T11‐UV TNBC cells were provided by Dr. Charles M. Perou (LCCC) and cultured in RPMI medium (Gibco 11965‐092) supplemented with 5% FBS, 1% penicillin–streptomycin and puromycin.

### 2.3 Preparation and characterization of POx micelles

The POx micelles of SB, LY, PTX, and LY/PTX (4/8), SB/PTX (5.2/8), LY/PTX (5.2/8) were prepared using the thin‐film hydration as recently described.^17^ Both the drugs and polymer were dissolved in ethanol, which served as a common solvent. The details of the evaporation and hydration conditions for each formulation are mentioned in Table S1. The drug loading into micelles was measured using reversed‐phase high‐pressure liquid chromatography (HPLC) on an Agilent 1200 HPLC system under isocratic elution mode with a mobile phase of acetonitrile (ACN)/water 70/30 (v/v) containing 0.1% trifluoroacetic acid operated at a flow rate of 1 mL/min. A Nucleosil C18, 5 μm particle size column (*L* × inner diameter [ID] 25 cm × 4.6 mm) was used, and the column temperature was set to 40°C. The micelle samples were diluted 50‐fold in ACN/water 50/50 (v/v), and an injection volume of 10 μL was used. The drug loading capacity of the POx formulations was measured as described previously. Furthermore, the size distribution of the formulations was determined using dynamic light scattering (DLS) on a Zetasizer Nano ZS (Malvern Instruments Ltd, UK). The samples were diluted 10‐fold in saline, and three measurements were recorded for each sample at 25°C. POx micelles were also characterized by transmission electron microscopy (TEM). The drug‐loaded micelles were diluted 10‐fold in distilled water to a final POx concentration of 2 mg/mL, dropped onto a TEM grid (Ted Pella Inc, Carbon Type‐B, 200 mesh, copper), allowed to dry, and then stained for 2 min with 1% uranyl acetate before TEM imaging. The Thermo Scientific Talos F200X scanning/transmission electron microscope (accelerating voltage of 200 kV) was used for image acquisition.

### 2.4 In vitro TGFβi release kinetics

The formulations were prepared as described in Table S1. After thin film hydration, formulations were diluted to 100 μg/mL active pharmaceutical ingredient for single drug and to 100 μg/mL PTX for combination drug release studies. Then, 100 μL of sample was added to dialysis devices (3.5 kDa MWCO, Thermo Fisher Product# 69552) for 1‐, 2‐, 6‐, and 24‐h time points (*N* = 3). An aliquot of initial dilution was reserved for 0‐h analysis (0% drug release). To 500 mL of phosphate buffered saline (PBS), 25 mL of FBS was added to prepare 5% FBS release medium. Next, 100 mL of release medium, equilibrated to 37°C, was added to a 500‐mL beaker. In a flotation device, 12 samples (4 time points × *N* = 3) for each formulation were added to the beaker and covered with foil to prevent evaporation. The beaker was shaken gently at 37°C for the duration of the study. At each specified time point, samples were completely removed from their dialysis devices, frozen in liquid nitrogen, and lyophilized overnight. Sample was resuspended in 50:50 ACN:water, centrifuged at 10,000*g* for 8 min, then added to HPLC vial. HPLC was run on Agilent 1200 series HPLC using a 250‐mm C18 Reversed‐Phase Column from Supelco. Analysis details for individual API in Table 1.

**TABLE 1:** HPLC analysis conditions for different active pharmaceutical ingredients.

Drug	Mobile phase (ACN:water)	UV analysis wavelength (nm)	Retention time (min)
Paclitaxel	60:40	227	6.2
SB525334	60:40	270	3.6
LY2109761	60:40	270	2.2

Abbreviation: ACN, acetonitrile.

### 2.5 In vitro TGFβ inhibition

The NIH‐3T3 cells were seeded in a 12‐well plate and grown overnight. The media was replaced after 24 h with fresh media containing 2.5 ng/mL TGFβ1 ligand (R&D Systems), with or without 5 μM TGFβi (SB‐POx, unformulated SB in dimethyl sulfoxide (DMSO) [SB], LY‐POx, unformulated LY in DMSO [LY]). Saline or vehicle (POx only or DMSO) were used as controls. Two hours after treatment, the cells were washed with PBS and lysed with radioimmunoprecipitation (RIPA) buffer (EMD Millipore) containing phosphatase and protease inhibitor cocktail (Sigma) and frozen overnight. The samples were thawed the next day, subjected to intermittent sonication, and centrifuged at 17,000*g* for 10 min at 4°C. The supernatant containing the whole cell lysate was collected for further analysis. The total protein concentration in the samples was measured using the Pierce BCA Protein Assay kit (Thermo Scientific). ProteinSimple Wes, a capillary‐based western blotting system, was used to assess the expression of phosphorylated Smad2/3 (p‐Smad2/3). The housekeeping protein was β‐actin. The antibodies were titrated to determine the optimal concentration with the least non‐specific binding. Table S2 contains information on the primary antibody clone, concentration, and lysate concentration. The anti‐rabbit secondary antibody and all other reagents were obtained from ProteinSimple. The NIH‐3T3 cells were treated with TGFβ1 with or without TGFβi in the same manner as in the western blot experiment. Cells not stimulated with TGFβ1 were used as unstimulated control. One hour after treatment, the cells were washed with PBS, detached with 100 μL of TrypLE Express (Gibco) treatment for 2 min, and neutralized with 1 mL medium. The cell suspension was then added to 10 volumes (10 mL) of 1× Lyse/Fix buffer (BD Phosphoflow) prewarmed to 37°C and incubated for 12 min at that temperature. The cell suspension was centrifuged at 600*g* for 7 min, and the supernatant was removed, leaving about 50 μL residual volume. The cell pellet was washed with 10 mL of BD stain buffer to remove traces of Lyse/Fix buffer and centrifuged again. After that, the cells were permeabilized by resuspending the cell pellet in 1 mL of pre‐chilled Perm Buffer III (BD Phosphoflow) and incubated on ice for 30 min. The cells were pelleted and washed three times with the BD stain buffer and resuspended at a final concentration of 10 M cells/mL with BD stain buffer before adding Fc block antibody (TruStain; diluted 50‐fold). Following a 15‐min incubation, PE anti‐p‐Smad2/3 antibody (see Table S2 for antibody information) was directly added to the cell suspension, and the cells were incubated for 60 min at room temperature (protected from light). A small percentage of cells were set aside as unstained control (USC) and did not receive the antibody stain. Following antibody staining, the cells were pelleted and washed twice with BD stain buffer, resuspended with 200 μL BD stain buffer, and mixed thoroughly before being analyzed on Attune NxT flow cytometer (Thermo Fisher).

### 2.6 Cytotoxicity

Eight thousand 4T1 cells were seeded per well in 96‐well plates and allowed to grow overnight. The following day, SB‐POx, LY‐POx, and PTX‐POx were serially diluted in the cell culture medium at fivefold increments, and nine different concentrations of each drug ranging from 0.256 nM to 100 μM were tested on cells. Wells that only received the media served as control. Twenty‐four hours following treatment, the cell viability was determined using the Dojindo Cell Counting Kit‐8 using the manufacturer's instructions. The half‐maximal inhibitory concentration (IC_50_) was calculated using the GraphPad Prism 9 software.

### 2.7 Tumor inhibition

Animal studies were conducted in accordance with the University of North Carolina at Chapel Hill Institutional Animal Care and Use Committee (IACUC) guidelines under approved IACUC IDs 19‐175.0‐C and 21‐220.0‐C (PI: Alexander V. Kabanov).

Animal studies were conducted in accordance with the University of North Carolina at Chapel Hill Institutional Animal Care and Use Committee guidelines. One million 4T1 cells (in 100 μL of 1:1 mix of Hank's balanced salt solution and BD Matrigel) were injected into the fourth left inguinal mammary fat pad of 8‐week‐old female BALB/c mice. For the T11‐Apobec and T11‐UV TNBC models, 0.1 million cells (in 100 μL of 1:1 mix of PBS and Cultrex Basement Membrane Extract [Type 3, R&D systems]) were injected into the fourth mammary fat pad of 7–8 weeks old female BALB/c mice. Treatments were initiated 7 days after tumor inoculation. A depilatory cream was used to remove the fur at the tumor site to enable accurate measurement of the tumor dimensions. Mouse survival, body weight changes, and tumor growth were monitored and recorded every other day (eod). The tumors were measured using vernier calipers, and the tumor volume was calculated as *V* = ½ × *L* × *W* ^2^. The tumor growth inhibition (TGI) was calculated as follows: TGI = (1 − mean final tumor volume of treatment group/mean final tumor volume of the control group) * 100. Animals that displayed stalled tumor growth attributable to unsuccessful tumor inoculation were excluded from the study. Animals exhibiting signs of distress such as hunched posture, ruffled fur, reduced mobility, and greater than 15% weight loss were euthanized via carbon dioxide intoxication. For all animal studies, a power analysis was conducted to confirm that all studies were adequately powered by calculating the differences in means by time points and treatment (threshold 80%) (see “Power analysis of animal studies” in Supporting Information).

### 2.8 Treatment regimens

**Varying TGFβi dosing frequencies.** Mice were randomized into groups of four and were treated as follows: (i) normal saline intravenously (i.v.) four times over 2 weeks (Th, M, Th, M) and intraperitoneally (i.p.) daily (Th‐M) for 2 weeks; (ii) 32 mg/kg SB‐POx i.p. daily (Th‐M) for 2 weeks; (iii) 32 mg/kg SB‐POx i.p. eod (Th, Sat, M) for 2 weeks; (iv) 75 mg/kg PTX‐POx i.v. four times over 2 weeks (Th, M, Th, M); (v) combination of (ii) and (iv); (vi) combination of (iii) and (iv).

**Varying TGFβi dose and comparison of i.v. versus i.p.** Mice were randomized into groups of four and were treated as follows: (i) normal saline i.v. four times over 2 weeks (Th, M, Th, M) and i.p. eod (Th, Sat, M) for 2 weeks; (ii) 50 mg/kg SB‐POx i.p. eod (Th, Sat, M) for 2 weeks; (iii) 50 mg/kg LY‐POx i.p. eod (Th, Sat, M) for 2 weeks; (iv) 75 mg/kg PTX‐POx i.v. four times over 2 weeks (Th, M, Th, M); (v) combination of (ii) and (iv); (vi) combination of (iii) and (iv); (vii) SB/PTX‐POx (5.2/8) i.v. four times over 2 weeks (Th, M, Th, M); (viii) LY/PTX‐POx (5.2/8) i.v. four times over 2 weeks (Th, M, Th, M); (ix) LY/PTX‐POx (4/8) i.v. four times over 2 weeks (Th, M, Th, M).

**Oral dosing of TGFβi.** Mice were randomized into groups of five and were treated as follows: (i) normal saline i.v. four times over 2 weeks (Th, M, Th, M) and via oral gavage (o.g.) eod (Th, Sat, M) for 2 weeks; (ii) 50 mg/kg SB‐POx o.g. eod (Th, Sat, M) for 2 weeks; (iii) 50 mg/kg LY‐POx via o.g. eod (Th, Sat, M) for 2 weeks; (iv) 75 mg/kg PTX‐POx (injected i.v. once every 4 days for 2 weeks); (v) combination of (ii) and (iv); (vi) combination of (iii) and (iv). In addition to the POx formulations, SB and LY were also administered in the form of a suspension composed of 0.5% sodium carboxymethylcellulose (NaCMC) and 0.25% Tween 80 as the control formulation.

### 2.9 Micro‐computed tomography

Animals from three groups (Saline [i.p. and i.v.], SB‐POx 50 mg/kg [i.p.], and SB‐POx 50 mg/kg [i.p.] and PTX‐POx 75 mg/kg [i.v.]) were chosen for whole‐body micro‐computed tomography (micro‐CT). The animals were anesthetized in an inhalation induction chamber (3% isoflurane in oxygen) and placed in the micro‐CT chamber in the supine position (anesthesia maintained at 2.5% isoflurane). The images were acquired on a PerkinElmer Quantum‐GX2 Imaging System (PerkinElmer, Inc., Waltham, MA, USA) with a 120 μm voxel size using the following scan settings: x‐ray tube voltage of 90 kV, current of 88 μA, Cu (0.06 mm) + Al (0.5 mm) filter, 4‐min scan duration, and a 60‐mm field of view. The scans were subjected to the retrospective lung‐gating protocol, creating two datasets corresponding to the inspiration and expiration phases of the breathing cycle. All datasets were calibrated to the Hounsfield scale. The image sets from the inspiration phase were used for analysis. A qualitative inspection was performed for each dataset to determine the presence of any metastatic lesions or tumors. The total lung volume was then segmented for each animal using a uniform threshold of −400 Hounsfield units (HU) to capture the lung tissue while excluding the vasculature. The mean lung tissue density (HU) over the entire volume was measured and compared across all study groups. Differences between groups were used to further determine the presence of potential lesions. All analyses were performed using Analyze 14.0 software (AnalyzeDirect, Inc., Overland Park, KS USA).

### 2.10 Histology

The resected lungs were fixed in 10% formaldehyde buffered solution for ≥24 h, followed by routine serial dehydration and paraffin embedding. The lungs were sectioned to 5 μm, stained with routine hematoxylin–eosin, and scanned using the Aperio AT2 digital scanner (2021 Leica Biosystems Imaging, Inc., Deer Park, IL, USA) at 20× magnification. The micrometastases in the lung were quantified using an image analysis algorithm. A boarded veterinary pathologist working in the UNC PSC used Definiens Architect XD 64 version 2.7.0.60765 to identify tumors in the sections, including those within and adjacent to lung parenchyma (presumed tumors arising from the thoracic lymph nodes). First, tissue (ROIs) on each slide was automatically detected using 221 brightness and 3.6 homogeneity thresholds and a minimum tissue size of 150,000 μm^2^. The two tissue sections on the slide were randomly assigned region of interest (ROI) labels “tissue 1” or “tissue 2” by the computer software. Next, large vessels, bronchi, and thymus were manually excluded from the ROIs. Examples of lung, glass (alveolar spaces), blood (erythrocytes in large vessels or alveolar spaces), and tumor were provided to the analysis software, which categorized the remaining lung sections into the appropriate tissue category. The resulting tumor area (μm^2^) was then divided by the lung area (μm^2^) for each ROI. Values were presented as tumor percent of the lung. The algorithm was validated by a randomized, semi‐quantitative assessment of the relative percent tumor burden by the same veterinary pathologist blinded to treatment groups.

### 2.11 Statistical analysis

GraphPad versions 8.4.3 and 9 were used for statistical analyses in this study. Numerical results are expressed as mean ± standard deviation in figures and tables. To evaluate differences in group means, we employed one‐way analysis of variance (ANOVA) (GraphPad 9). For comparison of the means of tumor volume between different treatments and timepoints, a two‐way ANOVA was used (GraphPad 8.4.3). We considered *p*‐values <0.05 evidence of differences in group means. The following symbols are presented in the figures to represent the significance level: *p < 0.05, **p < 0.01, ***p < 0.001, and ****p < 0.0001, ns (not significant).

### 2.12 Genomic data processing and visualization

Upper quartile normalized mRNA sequencing data from Hollern et al.^19^ (GEO: GSE124821) and from Lim et al.^20^ (GEO: GSE223630) were merged to a combined dataset of 232 individual mouse mammary tumors and normal mammary gland controls. Tumor models that contained less than three biological replicates were filtered out prior to log2 transformation and median centering by gene. Genes of interest were plotted by tumor model using the ggplot2 package (Version 3.3.6).

### 2.13 Data availability

**Table:** 

Raw and normalized data; RNA‐seq	Hollern et al.^19^	GEO: GSE124821
Raw and normalized data; RNA‐seq	Lim et al.^20^	GEO: GSE223630

## 3 RESULTS

### 3.1 Characterization of SB and LY containing PMs

**FIGURE 1:**
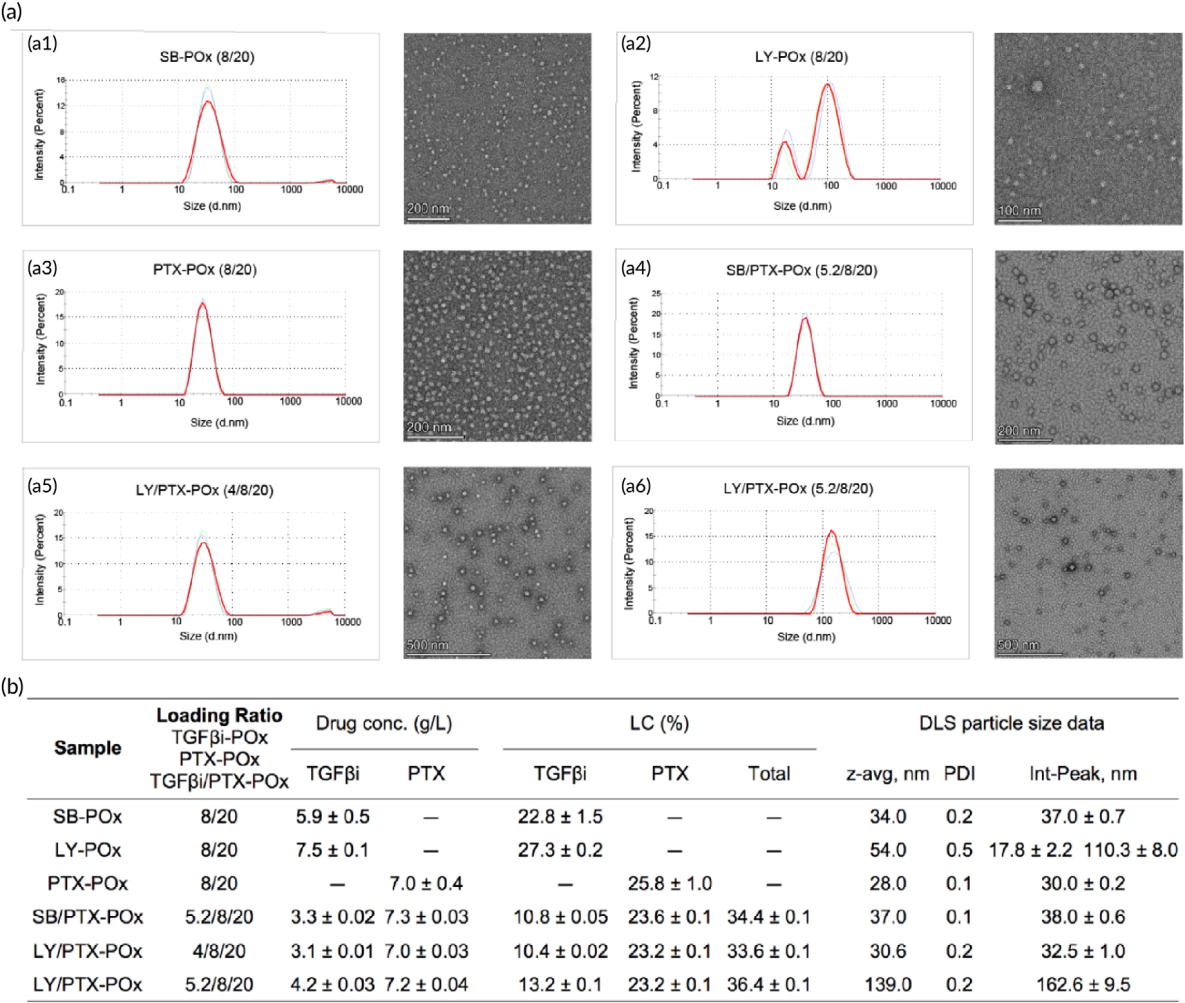
Characterization of single TGFβi and co‐loaded TGFβi/PTX in POx micelles by DLS and TEM. (a) DLS intensity size distributions and the corresponding TEM images of the nanoassemblies formed in drug‐loaded PMs of various compositions (a1) SB‐POx (8/20); (a2) LY‐POx (8/20); (a3) PTX‐POx (8/20); (a4) SB/PTX‐POx (5.2/8/20); (a5) LY/PTX‐POx (4/8/20); (a6) LY/PTX‐POx (5.2/8/20). The numbers in the brackets represent the mass ratios TGFβi/POx, PTX/POx, or TGFβi/PTX/POx. (b) Drug concentration, loading capacity (LC [%] = *M*_drug_/[*M*_drug_ + *M*_excipient_] × 100 [%]) and DLS characteristics (*z*‐average hydrodynamic diameter, PDI, diameters at intensity size distribution maxima) of the formulations presented in (a). Drug concentration in the solution was measured by HPLC. Data represent mean ± SE. For DLS measurements, the samples prepared at POx 20 mg/mL were diluted 10 times. DLS, dynamic light scattering; PDI, polydispersity index; PM, polymeric micelles; POx, poly(2‐oxazoline); PTX, paclitaxel; TEM, transmission electron microscopy; TGFβi, transforming growth factor beta inhibitors.

The POx PMs loaded with SB at 8/20 drug/polymer mass ratio were small with a reasonably narrow size distribution as evident from DLS (34 nm, PDI 0.17) and had a somewhat asymmetrical but compact shape based on TEM (Figures 1 and S1). The PTX‐containing PMs were also small and even more uniform (28 nm; PDI 0.07) which is consistent with our previous publication.^21^ Co‐loading of these two drugs in PMs at 5.2/8/20 SB/PTX/POx mass ratio produced uniform particles (37 nm; PDI 0.07) as well. On the other hand, PMs loaded with LY had a bimodal size distribution, with a smaller peak at around 18 nm and a larger peak at 110 nm (Figures 1 and S1). The mixture of small and large particles was also visible in TEM images. The co‐loaded LY/PTX‐POx were prepared at two mass ratios LY/PTX/POx 5.2/8/20 and 4/8/20. Of the two formulations, one (4/8/20) formed small micelles (30.6 nm; PDI 0.18) that had a nearly spherical shape (Figures 1 and S1). The other (5.2/8/20) formed relatively large particles (~140 nm; PDI 0.19) that appeared to be a mixture of separate and flocculated spheres, some of which may have further coalesced into the worm‐like structures (Figures 1 and S1).

### 3.2 Drug release kinetics of single and combination TGFβi and/or PTX PMs

SB and LY single‐agent TGFβi PMs exhibited a faster release of the API than single‐agent PTX‐POx (Figure S2). SB/PTX‐POx combination PMs released SB faster than PTX for all time points measured. Despite its slower release relative to SB, PTX from the combination PMs still released faster than PTX from single‐agent PTX‐POx for the first 4 h, after which it appeared to slow down relative to PTX‐POx (Figure S2A). In the LY/PTX‐POx combination PMs too, LY released faster than PTX and unlike SB/PTX‐POx, PTX from LY/PTX‐POx released faster than single‐agent PTX‐POx (Figure S2B). Compared to single‐agent TGFβi PMs, SB released faster from SB/PTX‐POx, whereas LY appeared to have a slightly slower release from LY/PTX‐POx. The differences in the release profiles of single‐agent and combination drug PMs can be explained on the basis of drug–drug interactions in the micelle core that ultimately impacts drug retention in the micelle, a phenomenon that has been previously reported for POx micelles.^22^

### 3.3 TGFβ inhibition activity of SB and LY and in vitro cytotoxicity of SB, LY, and PTX

**FIGURE 2:**
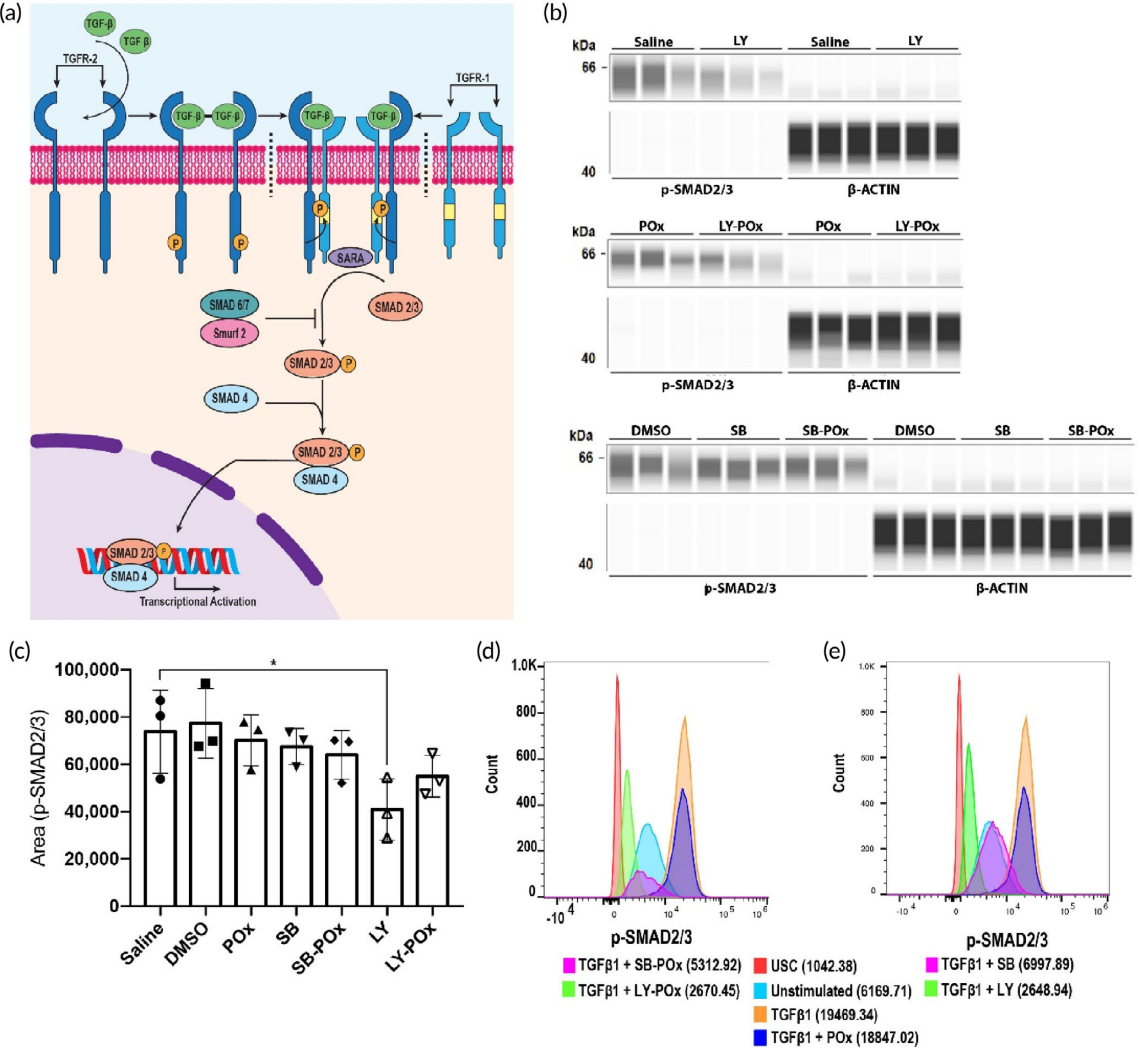
TGFβi treatment suppresses TGFβ signaling in NIH‐3T3 fibroblasts. (a) Schematic of canonical TGFβ signaling. (b) Capillary‐based western blot analysis of expression of p‐SMAD2/3 (~62 kDa) and housekeeping protein, β‐actin (~48 kDa) in NIH‐3T3 cells treated with TGFβi in POx PMs (SB‐POx [8/20] and LY‐POx [8/20]) or dissolved in DMSO. Control groups were treated with the same amounts of POx or DMSO. The images were generated using Compass for Simple Western (version 6.0). (c) The quantification of p‐SMAD2/3 bands in (b). *p < 0.05 computed by one‐way ANOVA with Tukey's multiple comparisons test. (d, e) The p‐SMAD2/3 expression analyzed by flow cytometry in NIH‐3T3 cells treated with TGFβi (d) formulated in POx PMs or (e) dissolved in DMSO. The (d) and (e) panels were run in the same experiment and split for the clarity of presentation to avoid overlap between the free and micelle‐formulated drug histograms. The panels have common histograms for USC, unstimulated, TGFβ‐stimulated controls. The mean florescence intensities for (d) and (e) are included in the parentheses. ANOVA, analysis of variance; PM, polymeric micelles; POx, poly(2‐oxazoline); TGFβ, transforming growth factor beta; TGFβi, TGFβ inhibitors; USC, unstained control.

TGFβ modulates ECM remodeling of the tumor microenvironment (TME) by activating TGFβ signaling in stromal cells, particularly fibroblasts, which engage in pro‐fibrotic responses.^23^ Phosphorylation of SMAD2 and SMAD3 are hallmarks of TGFβ signaling (Figure 2a). We used p‐SMAD2/3 expression levels as a marker for assessment of the inhibitory effect of our drugs on TGFβ signaling in TGFβ‐stimulated NIH‐3T3 fibroblasts. The western blot and flow cytometry analysis revealed p‐SMAD2/3 attenuation in groups treated with TGFβi relative to control groups. The TGFβ inhibition appeared to be more pronounced in groups treated with LY than SB, although the western blot quantification presented a trend but no significant difference between these groups (Figure 2b,c). However, the flow cytometry also corroborates the conclusion that LY is more potent than SB (Figure 2d,e). This difference may be explained by the ability of LY to inhibit both TGFR‐I and TGFR‐II, whereas SB inhibits only TGFR‐I.^24^ Both vehicles, DMSO and POx, had a negligible effect on the p‐SMAD2/3 levels. Because of the dose‐dependent cytotoxicity of PTX (IC_50_ 0.7 nM, Figure S3), we did not include this drug in the TGFβ inhibition study. Notably, SB and LY were substantially less toxic than PTX at concentrations ranging from 0.256 nM to 100 μM (Figure S3).

### 3.4 Evaluation of TGFβi and their combinations with PTX in the 4T1 immune‐competent animal model of TNBC

**FIGURE 3:**
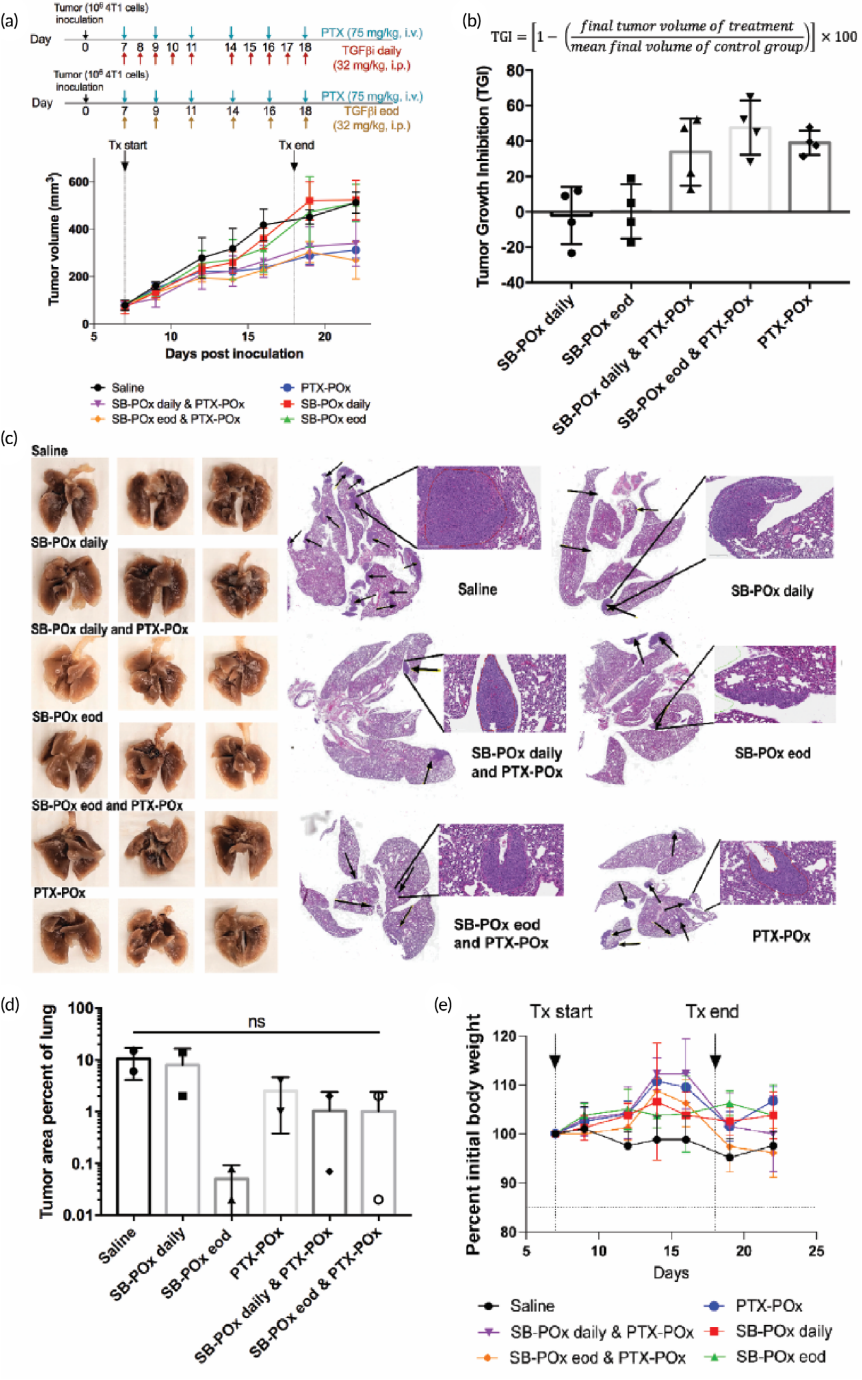
TGFβ inhibition synergizes with chemotherapy to inhibit primary tumor growth and lung metastases in 4T1 TNBC tumor‐bearing mice (*n* = 3 ~ 4). (a) Tumor growth curves following treatments with single‐drug SB‐POx (8/20) i.p., single‐drug PTX‐POx (8/20) i.v., or separately administered combinations SB‐POx (8/20) i.p. and PTX‐POx (8/20) i.v. using different SB‐POx schedules (daily or eod) at 32 mg/kg. See Table S3 for the complete statistical comparison between all groups. (Statistical comparisons were computed by two‐way ANOVA with Tukey's multiple comparisons test.) (b) Percent tumor growth inhibition corresponding to tumor growth curves. See Table S4 for the complete statistical comparison between all groups (Statistical comparisons were computed by one‐way ANOVA with Tukey's multiple comparisons test.) (c) Images of lungs with metastatic nodules (left) and corresponding H&E stain images (right) with metastatic nodules indicated with black arrows from representative mice in each group. (d) Histopathological scoring of lung metastatic burden. Statistical comparisons were computed by one‐way ANOVA with Tukey's multiple comparisons test. (e) Body weight changes (percent of initial) in mice treated with TGFβi‐POx and/or PTX‐POx. ANOVA, analysis of variance; eod, every other day; i.p., intraperitoneal; i.v., intravenous; PDI, polydispersity index; POx, poly(2‐oxazoline); PTX, paclitaxel; TGFβ, transforming growth factor beta; TNBC, triple‐negative breast cancer.

It has been previously reported that TGFβ downregulation by TGFβi is sustained for only a few hours after the drug is cleared from circulation, suggesting that frequent injection of LY is necessary for the persistent TGFβ pathway suppression.^25^ Accordingly, we decided to test daily versus eod i.p. dosing regimens for one formulation of TGFβi (SB‐POx). To this end, we used the 4T1 immune‐competent mouse model of TNBC, which is highly metastatic, therefore allowing for the testing of the treatment effectively both on primary as well as metastatic tumor burden. The tumor inhibition effect of SB‐POx at 32 mg/kg with respect to the primary tumor was not significantly different between the two regimens (Figure 3a). Of note, i.v. administration of PTX‐POx alone at 75 mg/kg suppressed the primary tumor growth relative to the control (Figure 3a,b). The tumor inhibition of the combination of SB‐POx (i.p.) and PTX‐POx (i.v.) did not differ significantly from that of PTX‐POx (Figure 3a,b). As far as the lung macrometastase and micrometastase were concerned, the differences between any of these groups were not significant. However, there was a trend for a decrease in the metastatic spread for PTX‐POx‐treated mice and even more so for SB‐POx using the eod regimen (Figure 3c,d). The animals maintained their weight during the course of the treatments (Figure 3e).

**FIGURE 4:**
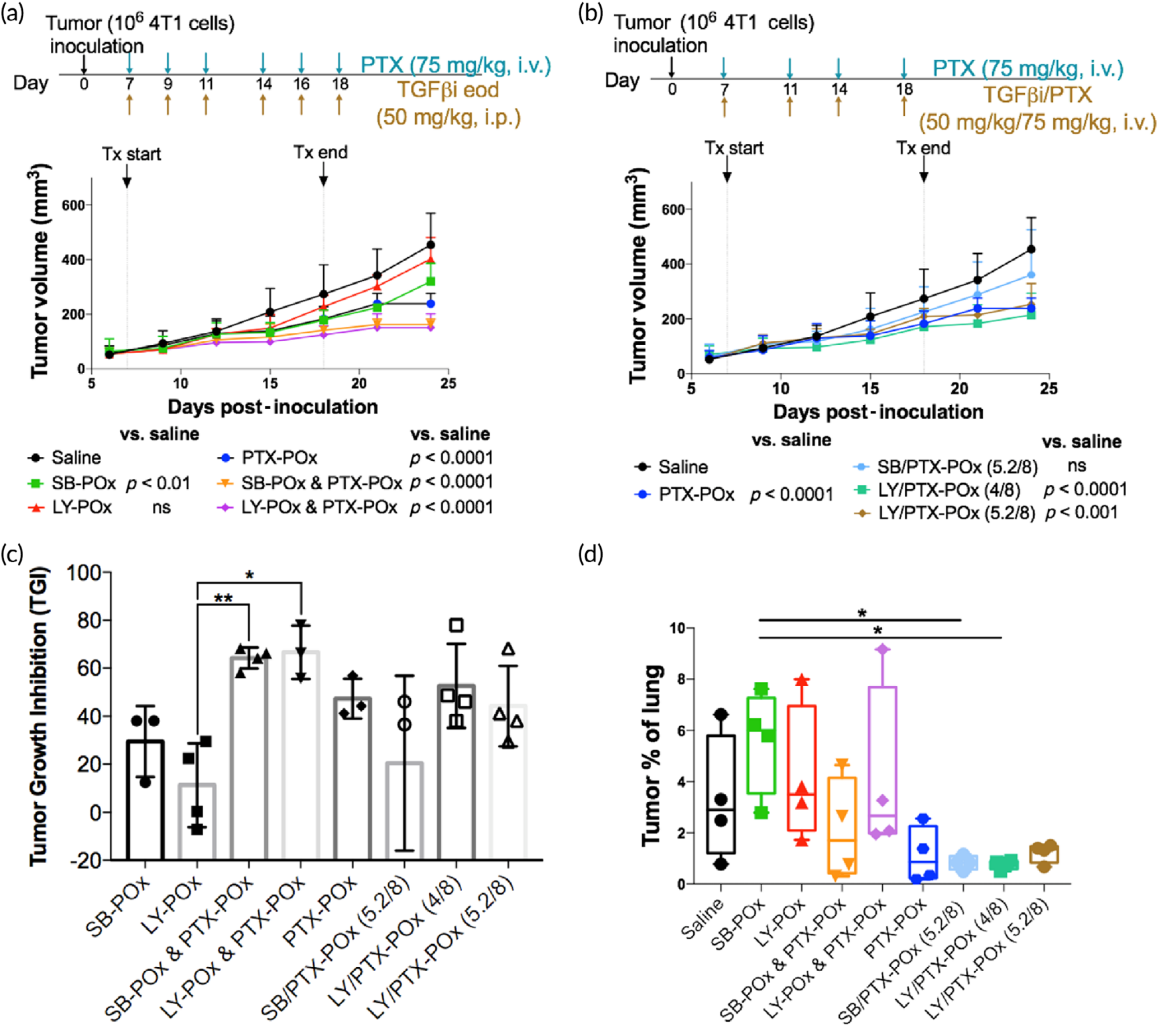
Tumor growth inhibition with i.p. versus i.v. routes of TGFβi delivery (*n* = 3–4). (a) Tumor growth curves in 4T1 tumor‐bearing mice treated with either single drug or separately administered TGFβi and PTX combinations, or (b) co‐loaded micelles of TGFβi and PTX (common saline and PTX‐POx groups were used for experiments (a) and (b) since the experiments were conducted in parallel); see Table S5 for the complete statistical comparison between all groups. (Statistical comparisons were computed by two‐way ANOVA with Tukey's multiple comparisons test.). (c) Percent tumor growth inhibition by treatments corresponding to (a) and (b). *p < 0.05 and **p < 0.01 computed by one‐way ANOVA with Tukey's multiple comparisons test. (d) Histopathological scoring of lung metastatic burden. Data represent mean ± SD. *N* = 2 with duplicate sections. *p < 0.05 computed by one‐way ANOVA with Tukey's post hoc test. ANOVA, analysis of variance; eod, every other day; i.p., intraperitoneal; i.v., intravenous; POx, poly(2‐oxazoline); PTX, paclitaxel; TGFβi, transforming growth factor beta inhibitor.

After determining that the eod dosing regimen for TGFβi was as effective as daily dosing, we decided to adhere to it and increased the TGFβi dose to 50 mg/kg for both SB and LY. This improved the performance of SB‐POx alone, which slowed down tumor growth relative to the saline control (Figure 4a). LY‐POx also slowed tumor growth but to a lesser extent than SB‐POx. Consistent with the previous experiment, i.v. PTX‐POx produced a notable antitumor effect. Moreover, we observed that the combinations of PTX‐POx with either TGFβi formulated in PMs provided some benefit over PTX‐POx alone (Figure 4c). There was a trend for an increase in antitumor effect when the PTX‐POx was combined with either LY‐POx or SB‐POx. Because the metastatic burden of the saline‐treated controls was at the low end, possibly due to the poor metastasis formation in the two animals selected for histopathology from this group, the effect of the treatments on the metastatic burden was confounded (Figures 4d and S4). Nonetheless, compared to the saline control in Figure 3d, the single‐drug treatments, as well as drug combinations, appeared to lower the metastatic burden in the lungs.

To reaffirm the extent of metastatic spread in the lungs of live animals in these groups, we examined the lungs of the tumor‐bearing animals for metastatic lesions by the in vivo micro‐CT. The lesions were small and below the resolution limit of this method (Figure S4A). Nonetheless, the overall lung tissue density appeared to decrease between saline, single SB‐POx, and combination SP‐POx and PTX‐POx groups as estimated by the HU (HU −594, −606, −636, respectively; Figure S4B). Although these differences were minor and not significant, they suggest a trend for decreasing metastatic nodules. The animal body weight was maintained during the single‐drug treatments. It decreased by ~13% during the combination drug treatments, although even in this case, it still stayed within the 15% cut‐off limit by the end of the study (Figure S4C).

Since the co‐loading of several drugs in PMs previously resulted in considerable treatment benefits,^22^ we examined the co‐loading approach for the combination of TGFβi and PTX. Here, we wanted to see if a co‐delivery of these agents in the same nanocarrier provided any benefit over separate administration of TGFβi and PTX at the same drug dose. The two examined co‐loaded drug formulations, LY/PTX‐POx (5.2/8/20) and LY/PTX‐POx (4/8/20), administered i.v. displayed significant antitumor activity, while one, SB/PTX‐POx (5.2/8/20), was statistically not different from the saline control (Figure 4b). The tumor growth inhibition in these treatment groups did not exceed ca. 50% even with the most potent combinations (Figure 4c). None of these combinations surpassed the efficacy of single PTX‐POx, and none of them was as effective as the combination treatments with micellar TGFβi and PTX administered separately (Figure 4a,c). This could be the effect of the cumulative dose of TGFβi which was 200 mg/kg for co‐loaded drugs (four injections, i.v.) and 300 mg/kg for drugs administered separately (six injections, i.p.). Since frequent i.v. injections cannot be realized in mice due to the risk of damaging the tail vein with repeated needle punctures, the eod regimen for the co‐loaded micelles was not tested for the i.v. route. We also would like to point out that the co‐loading strategy appeared to have some effect against the metastatic spread in the lungs, as evidenced by the lower percentages of metastatic burden in these groups. However, none of the values were significantly different from the saline control (Figures 4d and S5).

**FIGURE 5:**
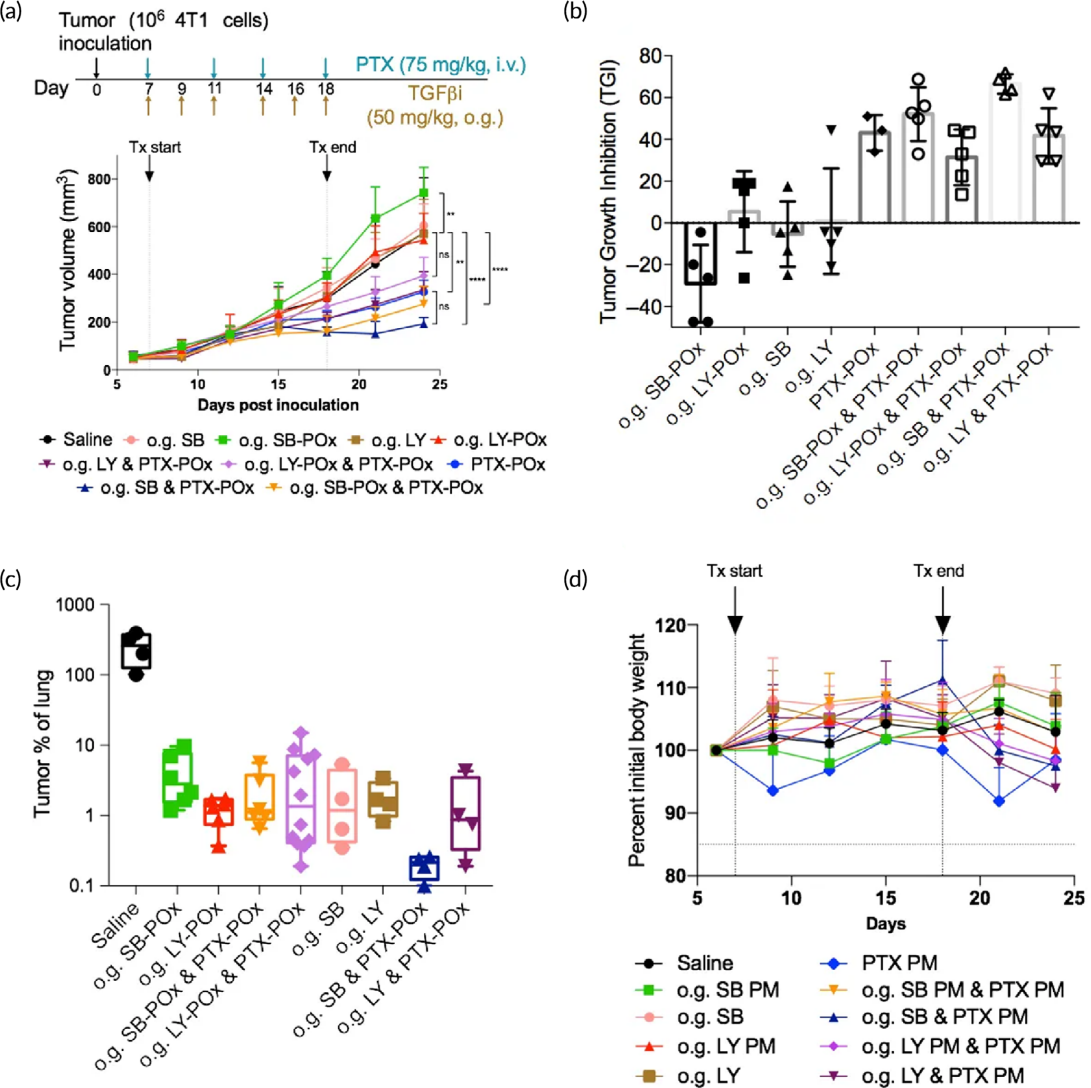
Tumor growth inhibition with oral route of TGFβi delivery (*n* = 5) (a) Tumor growth curves in 4T1 tumor‐bearing mice treated with orally delivered (o.g.) TGFβi formulated either in POx or 0.5% NaCMC and 0.25% Tween 80. Data represent mean ± SD. *N* = 4. **p < 0.003, ****p < 0.0001 computed by two‐way ANOVA with Tukey's post hoc test. See Table S6 for the complete statistical comparison between all groups. (Statistical comparisons were computed by two‐way ANOVA with Tukey's multiple comparisons test.). (b) Percent tumor growth inhibition by treatments corresponding to (a). See Table S7 for the complete statistical comparison between all groups. (Statistical comparisons were computed by one‐way ANOVA with Tukey's multiple comparisons test.) (c) Histopathological scoring of lung metastatic burden. Data represent mean ± SD. *N* = 2 with duplicate sections. ****p < 0.0001 computed by one‐way ANOVA with Tukey's post hoc test. (d) Body weight changes (percent of initial) in mice treated with TGFβi‐POx and/or PTX‐POx. ANOVA, analysis of variance; i.g., oral gavage; POx, poly(2‐oxazoline); PTX, paclitaxel; NaCMC, sodium carboxymethylcellulose; TGFβi, transforming growth factor beta inhibitor.

Finally, we tested the oral route of delivery for TGFβi using the eod regimen because both SB and LY are orally available.^26^, ^27^ In addition to POx formulations, SB and LY were also formulated in a conventional vehicle for oral administration (0.5% NaCMC and 0.25% Tween 80) to evaluate if POx micelles offer any advantage in terms of oral drug delivery. The results are presented in Figure 5. The single TGFβi was not effective when administered via o.g., independent of the formulation. In one case, o.g. SB‐POx, significantly increased tumor growth. (That contrasted sharply with the i.p. SB‐POx administered at the same dose [50 mg/kg, Figure 5a,b], which produced notable tumor inhibition.) The combination treatments with o.g. TGFβi and i.v. PTX‐POx, in all but one case, produced significant inhibition of the tumor growth compared to saline controls. However, they were not statistically different from the single i.v. PTX‐POx treatments. In this study, the o.g. TGFβi and i.v. PTX‐POx combination, as well as other treatments, produced a significant decrease in lung metastasis, as evidenced by the dramatic decrease in the metastatic lesions in the lungs of mice treated with the drugs when compared to the saline‐treated mice (Figures 5c and S6). Overall, when administered orally in combination treatments, the TGFβi in PMs performed as well as these agents in the conventional vehicle. Except for minor fluctuations, the animals in all the groups maintained their weight (Figure 5d). However, we need to point out that the animals receiving combination treatments with the oral TGFβi in the conventional vehicle were visually less active and appeared more dehydrated (displayed ruffled fur) than the groups receiving combinations with oral TGFβi in PMs.

**FIGURE 6:**
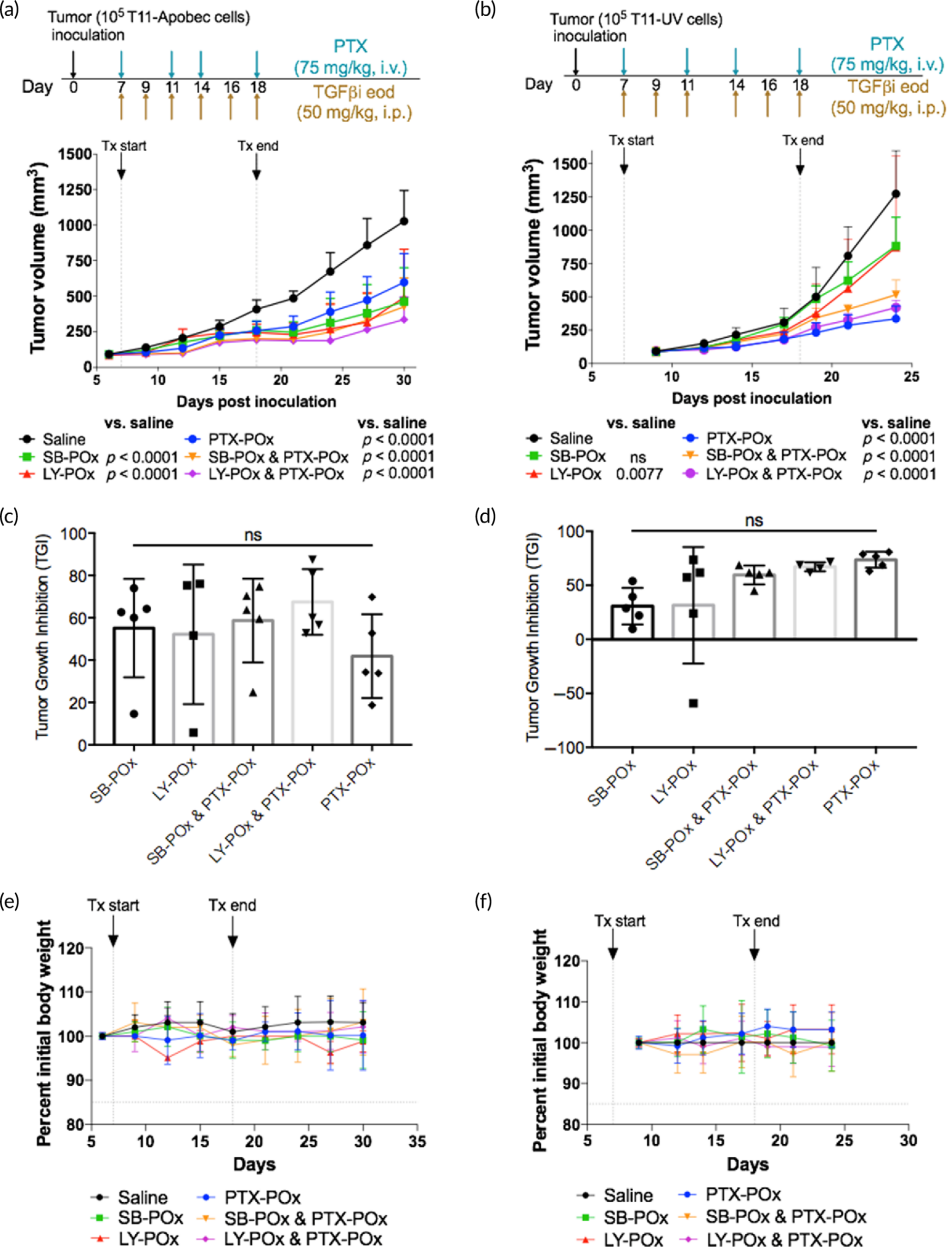
Differential sensitivity of Claudin‐low TNBC subtypes to TGFβi and PTX. Tumor growth curves following treatments with single‐drug SB‐POx (8/20) i.p., single‐drug PTX‐POx (8/20) i.v., or separately administered combinations SB‐POx (8/20) i.p. and PTX‐POx (8/20) i.v. in mice bearing (a) T11‐Apobec (*n* = 5) and (b) T‐11 UV tumors (*n* = 5). Data represent mean ± SD. *N* = 4. **p < 0.003, ****p < 0.0001 computed by two‐way ANOVA with Tukey's post hoc test. See Tables S8 and S9 for the complete statistical comparison between all groups. (c, d) Percent tumor growth inhibition by treatments corresponding to (a) and (b) as determined at the endpoint of tumor growth experiments. Statistical comparisons were computed by one‐way ANOVA with Tukey's multiple comparisons test. (e, f) Body weight changes (percent of initial) in mice treated with TGFβi‐POx and/or PTX‐POx. ANOVA, analysis of variance; i.p., intraperitoneal; i.v., intravenous; POx, poly(2‐oxazoline); PTX, paclitaxel; TGFβi, transforming growth factor beta inhibitor; TNBC, triple‐negative breast cancer.

To further explore the possibility of using the TGFβi and PTX PMs to treat other TNBC models, we screened an array of gene expression profiles of 19 transplantable syngeneic genetically engineered mouse (GEM) models of TNBC and two normal mammary gland controls available at UNC Lineberger Cancer Center and assessed them based on the relative expression of genes known to regulate both the canonical and non‐canonical TGFβ pathways (*Tgfbr1*, *Tgfbr2*, *Tgfb1*, *Tgfb2*, *Vim*, *Cd36*, *Egfr*, *Mapk1*, *Mapk3*, *Pik3ca*). As presented in Figure S7, our 4T1 model displays TGFβ signature genes (*Tgfbr1*, *Tgfb1*), EMT signature (*Vimentin* and *Mapk3*), and fibrotic signature (*Pik3ca*), and a low expression of fibroblast quiescence marker (*Cd36*). We selected two additional tumor models, T11‐UV and T11‐Apobec, that displayed a high expression of *Tgfb1* (both tumor models) and *Tgfb2* (T11‐UV), high expression of *Vimentin* (*Vim*) and fibrotic marker (*Pik3ca*), and low expression of *Cd36* and apoptosis regulator *Bcl2* (Figure S7). Both models are derived from T11 murine breast cancer that closely resembles the genomic phenotype and immune responses seen in patients with Claudin‐low breast cancer.^19^, ^28^, ^29^, ^30^ T11 cells (a *Trp53*−/− cell line) overexpressing *Apobec3* and T11 cells exposed to short‐wave UV exposure were transplanted into syngeneic hosts to generate T11‐Apobec and T11‐UV models, respectively.^19^ Interestingly, these models responded differently to our drug treatments. TGFβi SB‐POx or LY‐POx alone markedly inhibited the T11‐Apobec tumor growth (Figure 6a,c). The PTX‐POx alone had a significant but smaller tumor inhibition effect. The tumor growth regressed more significantly when treated with the combination of TGFβi‐POx and PTX‐POx compared to either of the treatments alone. In contrast, in the T11‐UV tumor, the response to the single‐drug TGFβi therapies SB‐POx or LY‐POx was considerably less than that observed in single‐drug PTX‐POx treatment (Figure 6b,d). In this regard, T11‐UV behaved somewhat similarly to the 4T1 tumor. The combinations of TGFβi and PTX PMs did not provide an added benefit in these tumors over the PTX‐POx treatment. All animals maintained their weights during the treatments (Figure 6e,f).

## 4 DISCUSSION

POx micelle nanoformulation technology demonstrates an extremely high loading of small molecules while requiring a significantly lesser amount of polymeric excipient than conventional vehicles.^31^ This translates to (1) high‐dose PTX delivery with few side effects^21^ and (2) co‐delivery of two drugs in a single micelle, thereby increasing the tumor distribution and antitumor effect of these drugs.^22^, ^32^ Our goal in this study was to evaluate whether this platform technology could improve treatment outcomes during chemo‐immunotherapy in the mouse models of TNBC. Here we combine PTX, one of the most active cytotoxic agents for TNBC medical management,^33^ with the agents capable of inhibiting the TGFβ signaling pathway.

The TGFβ cytokine family comprises three isoforms, TGFβ1, TGFβ2, and TGFβ3, two transmembrane serine/threonine receptors, TGFR‐I, TGFR‐II, and a coreceptor, TGFR‐III. The cytokines are homodimers that initiate the TGFβ signaling by binding to TGFR‐II, which is constitutively active and undergoes autophosphorylation. The phosphorylated TGFR‐II recruits TGFR‐I resulting in the formation of a heterotetrameric complex of TGFR‐I and TGFR‐II, following which TGFR‐II phosphorylates the serine residues of TGFR‐I.^34^ The signal is relayed downstream through the phosphorylation of receptor‐regulated SMADs (R‐SMADs: SMAD 1, SMAD 2, SMAD 3, SMAD 5, and SMAD 9) by TGFR‐I; subsequently, the phosphorylated R‐SMADs form a complex with co‐SMAD and the complex translocates to the nucleus and initiates gene transcription.^35^

Clinical studies of small molecule TGFβi involve their repeated oral administration, either twice or once daily, due to the short‐lived effect of p‐SMAD inhibition by these molecules.^36^ In the present study, we varied dosing routes and frequencies to test if i.p. administration of TGFβi improved its antitumor efficacy compared to o.g. The i.v. administration route is not suitable for repeated dosing of drugs and was not included in our design since it is closely approximated by the i.p. administration in rodents.^37^

Two small‐molecule TGFβi with different potencies for the kinase domain were studied in this work: (1) SB (GlaxoSmithKline), an imidazole derivative with an IC_50_ of 14.3 nM for TGFR‐I; and (2) LY (Eli Lilly), a pyrazole‐based dual inhibitor of TGFR‐I (Ki = 38 nM) and TGFR‐II (Ki = 300 nM).^24^ Both agents were tested in animal models (rodents), but neither advanced to human clinical evaluation, likely due to their poor solubility. To our knowledge, none of them has a format available for either i.v. or p.o. administration. We successfully reformulated these molecules in our POx PMs, resulting in highly loaded, small, and stable nanoparticles for SB and somewhat less stable but still useful nanoformat for LY. The resulting POx nanoformulations in the present study generally demonstrated safety and activity, which provides a basis for further translational development of these agents.

We used a well‐known 4T1 TNBC model in the initial phase of our work. Contradictory to previous literature that suggests 4T1 to be basal,^38^, ^39^, ^40^ recent work by our group identified 4T1 to be a transcriptomic hybrid between Claudin‐low and basal‐like, lacking clear gene expression features of both.^20^ The 4T1 tumors display an immune suppressive gene signature with upregulation of Th2, T‐regulatory, PD‐1, and CTLA4 signaling.^41^ As for the mutation signature, a multi‐omics study of 4T1 tumors reported mutation in the Trp53 and PIK3cg (catalytic subunit of PI3 kinase) genes. The study also revealed mutations in markers for metastasis, cell proliferation, and cancer‐related genes. The 4T1 model is highly aggressive and does not display a mutation in the BRCA genes.^41^ BRCA‐deficient basal‐like tumors are more sensitive to chemotherapy. Because TGFβ signaling promotes the development of T‐regulatory cells, which are key players in regulating immune tolerance, it strengthens the rationale for using TGFβi in this tumor to restore the tumor's suppressed immune surveillance.^42^

Since the i.p. route has a greater extent and rate of absorption than the oral route, we examined if daily dosing of TGFβi in PMs has any benefit compared to the eod dosing.^37^ The tumor inhibition study using the 4T1 TNBC model revealed that daily dosing of micellar SB‐POx was comparable to eod dosing with regard to the primary as well as metastatic tumor inhibition. Based on this, we proceeded with the eod dosing. Of the two agents, SB‐POx appeared to be more effective—at the higher dose, it has shown potency in inhibiting primary tumor growth. In contrast, LY‐POx had a modest effect on the primary tumor. Regarding suppression of lung metastases, a secondary endpoint in our study, we did not see definitive evidence of the superiority of any of our formulations. However, we observed at least a trend of improved suppression with several treatments, which provides the basis for further assessment of this matter using dedicated metastatic models. The effect of the TGFβi on the metastatic spread could be related to the suppression of the TGFβ‐mediated EMT program, which can reduce the invasive and metastatic capacity of the primary tumors.^43^

We further evaluated if the oral dosing of TGFβi in either a conventional vehicle or micelles could be more advantageous than systemic POx formulation. As a single drug, there was no benefit of oral dosing, and for one drug, SB, the oral dosing was even detrimental and increased tumor growth. In contrast, SB‐POx (i.p.) at the same dose produced significant tumor inhibition. The underperformance of the oral SB‐POx compared to the systemic dosing could be due to the poor oral absorption of the drug, resulting in its sub‐therapeutic levels in the tumor. The interspecies difference in the oral bioavailability of TGFβi has been previously reported, with considerably lower bioavailability in small animals compared to large animals.^44^ Furthermore, prolonged exposure to a low dose of TGFβi has been shown to result in the development of drug resistance.^25^ This could be attributed to the glycoprotein P (P‐gp) expression in the intestinal endothelium in the gut, which could be overcome by P‐gp inhibitors such as Tween 80 contained in the conventional vehicle NaCMC.^45^ However, Tween 80 is implicated in several side effects and, therefore, must be substituted by alternative, better‐tolerated formulations.^46^

Next, we proceeded with the combination therapy using TGFβi and PTX. First, we concluded that the co‐formulation of these agents in the same PM does not have any benefit compared to their separate administration because of the very different pharmacodynamics and treatment schedules of these drugs. The drugs administered separately at their optimal dosing schedules have shown superior tumor inhibition compared to co‐formulated drugs. Second, we clearly see that the combination treatments with two drugs surpass treatments with each single TGFβi in the 4T1 model. The combination treatments using the higher dose of TGFβi also appear to be more effective than the single PTX‐POx. For the combination treatments within the design of this study, we could not see any significant difference between the oral and systemic dosing of TGFβi PMs.

To further evaluate the broad applicability of our dosing approach, we tested the antitumor efficacy of TGFβi and PTX in POx PMs using our credentialed mouse models of TNBC. We selected two Claudin‐low TNBC models, T11‐Apobec and T11‐UV, based on the expression of genetic markers for TGFβ signaling and EMT. Most importantly, the combination treatments using these two drugs in our PMs format were uniformly effective across all three models, including the 4T1 model discussed above. However, we noted with interest that the T11‐Apobec and T11‐UV models showed different sensitivities to single‐drug treatments. Specifically, the T11‐UV tumor responded well to PTX‐POx but had lower responses to either of the TGFβi‐POx. Conversely, the T11‐Apobec tumor was highly responsive to each of the single TGFβi‐POx but was somewhat less responsive to PTX‐POx.

We would like to point out the differences in the mutation signatures and tumor mutation burden (TMB) of the T11‐Apobec and T11‐UV tumors, which are known to regulate response to treatments. The mutation signatures of cancers dictate their immunogenicity and, thus, response to treatments that stimulate immune surveillance, such as immune checkpoint blockade.^47^, ^48^ Apobec3 is a family of antiviral enzymes that are implicated in tumorigenesis. Tumors overexpressing *Apobec3* are less likely to respond to chemotherapy but are sensitized to immunotherapy.^49^ Moreover, *Apobec3G* expression correlates with the expression of TGFR1 and TGFβ1, one (TGFβ1) of which was found to be highly expressed in this model. In addition to Apobec3G regulating TGFβ1 signaling,^50^ the latter also regulates the former's expression,^51^ alluding to possible crosstalk between the pathways. Therefore, targeting the TGFβ pathway should also attenuate Apobec3‐mediated resistance of tumors to chemotherapy. The observed synergistic effect of the combination of TGFβ and PTX in these tumors in the present study further corroborates this.

The transcriptome evaluation of the Claudin‐low T11‐UV tumors revealed a decrease in the *Bcl2* gene expression compared to the normal mammary tissue. *Bcl2* is an apoptosis regulator in cells, and its expression negatively correlates with sensitivity to PTX.^52^ We noted that the gene expression of *Bcl2* in T11‐UV was even lower than in T11‐Apobec, which could explain the higher sensitivity of this tumor to PTX relative to T11‐Apobec. Also, T11‐UV has a higher mutation load than T11‐Apobec (TMB = 1783 and 374, respectively), and both are markedly higher than the parental cell line, T11 (TMB = 117).^19^ Higher TMB for many human tumors has been shown to correlate with higher levels of neoantigens and with increased survival after immune checkpoint inhibitor therapy.^53^, ^54^ Since TGFβ controls the regulation of immune tolerance^55^ and response to immunotherapy,^56^ higher TMB is expected to contribute to a higher sensitivity to the immunomodulating effects of TGFβi. However, that is not the case with T11‐UV. Thus, the differences in the responses of T11‐Apobec and T11‐UV to the single TGF‐beta inhibitors cannot be simply explained by TMB and may also depend on the differences in their tumor mutation signatures.

The use of TGFβi in combination with our high‐dose PTX in POx micelles may also be a “double‐punch” immunotherapeutic intervention against TNBC. PTX, in addition to its antimicrotubule activity, is also a Toll‐like receptor 4 (TLR4) agonist and was shown to activate pro‐inflammatory responses in macrophages.^57^ TLR4 gene is expressed in all three tumor models studied in the present work (Figure S7). Moreover, our prior study suggested that high‐dose PTX‐POx therapy can enhance immune cell death, repolarize tumor‐associated macrophages to a pro‐inflammatory M1‐like state, and increase T‐cell‐mediated immune responses in the tumor microenvironment in several TNBC tumors, including 4T1 and T11‐Apobec.^20^ This drug also produced long‐term immune memory in 4T1 tumors.^20^ Altogether, the activation of the TLR4 pathway by PTX in combination with TGFβ inhibition can further invigorate the immune tumor microenvironment across tumor models, which may explain the robust performance of the combination treatments. Future investigation delineating mutation signatures that favor better outcomes in response to the combination therapy of TGFβi and PTX could prove helpful in genetically defining treatment‐responsive tumor subgroups.

In summary, our study suggests that combination therapy with TGFβis and PTX in high‐capacity POx micelles provides a robust antitumor response in multiple TNBC subtype models. Therefore, we posit that the combination of TGFβi with PTX in high‐capacity PMs could be a good remedy for TNBC.

## AUTHOR CONTRIBUTIONS

**Natasha Vinod:** Conceptualization; data curation; investigation; methodology; writing – original draft; writing – review and editing. **Duhyeong Hwang:** Conceptualization; data curation; investigation; methodology; validation; writing – review and editing. **Sloane Christian Fussell:** Data curation; methodology; visualization. **Tyler Cannon Owens:** Data curation; methodology. **Olaoluwa Christopher Tofade:** Methodology. **Thad S. Benefield:** Validation; writing – original draft. **Sage Copling:** Methodology. **Jacob D. Ramsey:** Data curation; methodology; writing – review and editing. **Patrick D. Rädler:** Data curation; formal analysis; writing – review and editing. **Hannah M. Atkins:** Formal analysis; visualization; writing – review and editing. **Eric E. Livingston:** Formal analysis; visualization; writing – original draft. **J. Ashley Ezzell:** Methodology. **Marina Sokolsky‐Papkov:** Project administration; supervision. **Hong Yuan:** Investigation; supervision. **Charles M. Perou:** Funding acquisition; supervision. **Alexander V. Kabanov:** Funding acquisition; supervision; writing – review and editing.

## FUNDING INFORMATION

This work was partially supported by the National Cancer Institute (NCI) Alliance for Nanotechnology in Cancer (U54CA198999, Carolina Center of Cancer Nanotechnology Excellence), and NCI's grant R01CA264488 (to AVK) and the UNC Eshelman Institute for Innovation grant RX03912424 (PI Leaf Huang). Partial support was also provided by the NCI Breast SPORE program (P50‐CA058223), and RO1‐CA148761 (to CMP).

## CONFLICT OF INTEREST STATEMENT

A.V.K. is an inventor on patents pertinent to the subject matter of the present contribution, co‐founder, stockholder and director of DelAqua Pharmaceuticals Inc. having intent of commercial development of POx‐based drug formulations. A.V.K. is also a co‐founder, stockholder and director of SoftKemo Pharma Corp. and BendaRx Pharma Corp., which develop polymeric drug formulation and a blood cancer drug. M.S.P. discloses potential interest in DelAqua Pharmaceuticals Inc., SoftKemo Pharma Corp. and BendaRx Pharma Corp. as a spouse of a co‐founder. C.M.P is an equity stockholder and consultant of BioClassifier LLC; C.M.P is also listed as an inventor on patent applications for the Breast PAM50 Subtyping assay.

### PEER REVIEW

The peer review history for this article is available at https://www.webofscience.com/api/gateway/wos/peer-review/10.1002/btm2.10681.

## Supporting information


**Data S1.** Supplementary Information.

## Data Availability

At the latest upon publication, all cancer‐relevant nanoparticle data with characterizations from physicochemical (molecular weight, size, shape, solubility, etc.), in vitro (cytotoxicity, toxicity, etc.), and in vivo (imaging, efficacy, toxicology etc.) assays will be made available through the Cancer Nanotechnology Laboratory (caNanoLab) data portal. caNanoLab is a data‐sharing portal designed to facilitate information sharing in the biomedical nanotechnology research community to expedite and validate the use of nanotechnology in biomedicine. caNanoLab provides support for the annotation of nanomaterials with characterizations resulting from physico‐chemical, in vitro, and in vivo assays and the sharing of these characterizations and associated nanotechnology protocols in a secure fashion.

## References

[1] Sung H , Ferlay J , Siegel RL , et al. Global cancer statistics 2020: GLOBOCAN estimates of incidence and mortality worldwide for 36 cancers in 185 countries. CA Cancer J Clin. 2021;71(3):209‐249. 33538338 10.3322/caac.21660

[2] Prat A , Parker JS , Karginova O , et al. Phenotypic and molecular characterization of the claudin‐low intrinsic subtype of breast cancer. Breast Cancer Res. 2010;12(5):R68. 20813035 10.1186/bcr2635 PMC3096954

[3] Lehmann BD , Bauer JA , Chen X , et al. Identification of human triple‐negative breast cancer subtypes and preclinical models for selection of targeted therapies. J Clin Invest. 2011;121(7):2750‐2767. 21633166 10.1172/JCI45014 PMC3127435

[4] Marra A , Trapani D , Viale G , Criscitiello C , Curigliano G . Practical classification of triple‐negative breast cancer: intratumoral heterogeneity, mechanisms of drug resistance, and novel therapies. NPJ Breast Cancer. 2020;6:54. 33088912 10.1038/s41523-020-00197-2 PMC7568552

[5] Fornier M , Fumoleau P . The paradox of triple negative breast cancer: novel approaches to treatment. Breast J. 2012;18(1):41‐51. 22098334 10.1111/j.1524-4741.2011.01175.x

[6] Li CH , Karantza V , Aktan G , Lala M . Current treatment landscape for patients with locally recurrent inoperable or metastatic triple‐negative breast cancer: a systematic literature review. Breast Cancer Res. 2019;21:1‐14. 31842957 10.1186/s13058-019-1210-4 PMC6916124

[7] Lyons T . Targeted therapies for triple‐negative breast cancer. Curr Treat Options Oncol. 2019;20:1‐13. 10.1007/s11864-019-0682-x 31754897

[8] Kim D , Nam HJ . PARP inhibitors: Clinical limitations and recent attempts to overcome them. Int J Mol Sci. 2022;23(15):8412. 10.3390/ijms23158412 PMC9369301 35955544

[9] Block KI , Gyllenhaal C , Lowe L , et al. Designing a broad‐spectrum integrative approach for cancer prevention and treatment. Semin Cancer Biol. 2015;35:S276‐S304. 26590477 10.1016/j.semcancer.2015.09.007 PMC4819002

[10] Heeke AL , Tan AR . Checkpoint inhibitor therapy for metastatic triple‐negative breast cancer. Cancer Metastasis Rev. 2021;40(2):537‐547. 34101053 10.1007/s10555-021-09972-4 PMC8184866

[11] D'Abreo N , Adams S . Immune‐checkpoint inhibition for metastatic triple‐negative breast cancer: safety first? Nat Rev Clin Oncol. 2019;16(7):399‐400. 31053774 10.1038/s41571-019-0216-2

[12] Elliott RL , Blobe GC . Role of transforming growth factor beta in human cancer. J Clin Oncol. 2005;23(9):2078‐2093. 15774796 10.1200/JCO.2005.02.047

[13] Marincola Smith P , Means AL , Beauchamp RD . Immunomodulatory effects of TGF‐β family signaling within intestinal epithelial cells and carcinomas. Gastrointest Disord. 2019;1(2):24‐300. 10.3390/gidisord1020024 PMC8025874 33834163

[14] Nedeljkovic M , Damjanovic A . Mechanisms of chemotherapy resistance in triple‐negative breast cancer‐how we can rise to the challenge. Cells. 2019;8:957. 10.3390/cells8090957 PMC6770896 31443516

[15] Bellomo C , Caja L , Moustakas A . Transforming growth factor β as regulator of cancer stemness and metastasis. Br J Cancer. 2016;115(7):761‐769. 27537386 10.1038/bjc.2016.255 PMC5046208

[16] Bhola NE , Balko JM , Dugger TC , et al. TGF‐beta inhibition enhances chemotherapy action against triple‐negative breast cancer. J Clin Invest. 2013;123(3):1348‐1358. 23391723 10.1172/JCI65416 PMC3582135

[17] Vinod N , Hwang D , Azam SH , et al. Preparation and characterization of poly(2‐oxazoline) micelles for the solubilization and delivery of Water insoluble drugs. Bio Protoc. 2021;11(6):e3959. 10.21769/BioProtoc.3959 PMC8032494 33855119

[18] Luxenhofer R , Schulz A , Roques C , et al. Doubly amphiphilic poly(2‐oxazoline)s as high‐capacity delivery systems for hydrophobic drugs. Biomaterials. 2010;31(18):4972‐4979. 20346493 10.1016/j.biomaterials.2010.02.057 PMC2884201

[19] Hollern DP , Xu N , Thennavan A , et al. B cells and T follicular helper cells mediate response to checkpoint inhibitors in high mutation burden mouse models of breast cancer. Cell. 2019;179(5):1191‐1206 e21. 31730857 10.1016/j.cell.2019.10.028 PMC6911685

[20] Lim C , Hwang D , Yazdimamaghani M , et al. High‐dose paclitaxel and its combination with CSF1R inhibitor in polymeric micelles for chemoimmunotherapy of triple negative breast cancer. Nano Today. 2023;51:101884. 37484164 10.1016/j.nantod.2023.101884 PMC10357922

[21] He Z , Wan X , Schulz A , et al. A high capacity polymeric micelle of paclitaxel: implication of high dose drug therapy to safety and in vivo anti‐cancer activity. Biomaterials. 2016;101:296‐309. 27315213 10.1016/j.biomaterials.2016.06.002 PMC5035646

[22] Wan X , Beaudoin JJ , Vinod N , et al. Co‐delivery of paclitaxel and cisplatin in poly (2‐oxazoline) polymeric micelles: implications for drug loading, release, pharmacokinetics and outcome of ovarian and breast cancer treatments. Biomaterials. 2019;192:1‐14. 30415101 10.1016/j.biomaterials.2018.10.032 PMC6331221

[23] Shi X , Young CD , Zhou H , Wang X . Transforming growth factor‐beta signaling in fibrotic diseases and cancer‐associated fibroblasts. Biomolecules. 2020;10(12):1666. 10.3390/biom10121666 PMC7763058 33322749

[24] Wang H , Chen M , Sang X , et al. Development of small molecule inhibitors targeting TGF‐beta ligand and receptor: structures, mechanism, preclinical studies and clinical usage. Eur J Med Chem. 2020;191:112154. 32092587 10.1016/j.ejmech.2020.112154

[25] Connolly EC , Saunier EF , Quigley D , et al. Outgrowth of drug‐resistant carcinomas expressing markers of tumor aggression after long‐term TbetaRI/II kinase inhibition with LY2109761. Cancer Res. 2011;71(6):2339‐2349. 21282335 10.1158/0008-5472.CAN-10-2941 PMC3059399

[26] Heo JY , Do JY , Lho Y , Kim AY , Kim SW , Kang SH . TGF‐beta1 receptor inhibitor SB525334 attenuates the epithelial to mesenchymal transition of peritoneal mesothelial cells via the TGF‐beta1 signaling pathway. Biomedicine. 2021;9(7): 839. 10.3390/biomedicines9070839 PMC8301792 34356903

[27] Melisi D , Ishiyama S , Sclabas GM , et al. LY2109761, a novel transforming growth factor beta receptor type I and type II dual inhibitor, as a therapeutic approach to suppressing pancreatic cancer metastasis. Mol Cancer Ther. 2008;7(4):829‐840. 18413796 10.1158/1535-7163.MCT-07-0337 PMC3088432

[28] Hollern DP , Contreras CM , Dance‐Barnes S , et al. A mouse model featuring tissue‐specific deletion of p53 and Brca1 gives rise to mammary tumors with genomic and transcriptomic similarities to human basal‐like breast cancer. Breast Cancer Res Treat. 2019;174(1):143‐155. 30484104 10.1007/s10549-018-5061-y PMC6418066

[29] Pfefferle AD , Herschkowitz JI , Usary J , et al. Transcriptomic classification of genetically engineered mouse models of breast cancer identifies human subtype counterparts. Genome Biol. 2013;14(11):R125. 24220145 10.1186/gb-2013-14-11-r125 PMC4053990

[30] Pfefferle AD , Agrawal YN , Koboldt DC , et al. Genomic profiling of murine mammary tumors identifies potential personalized drug targets for p53‐deficient mammary cancers. Dis Model Mech. 2016;9(7):749‐757. 27149990 10.1242/dmm.025239 PMC4958311

[31] Hwang D , Ramsey JD , Kabanov AV . Polymeric micelles for the delivery of poorly soluble drugs: from nanoformulation to clinical approval. Adv Drug Deliv Rev. 2020;156:80‐118. 32980449 10.1016/j.addr.2020.09.009 PMC8173698

[32] Wan X , Min Y , Bludau H , et al. Drug combination synergy in worm‐like polymeric micelles improves treatment outcome for small cell and non‐small cell lung cancer. ACS Nano. 2018;12(3):2426‐2439. 29533606 10.1021/acsnano.7b07878 PMC5960350

[33] Schettini F , Giuliano M , De Placido S , Arpino G . Nab‐paclitaxel for the treatment of triple‐negative breast cancer: rationale, clinical data and future perspectives. Cancer Treat Rev. 2016;50:129‐141. 27665540 10.1016/j.ctrv.2016.09.004

[34] Poniatowski ŁA , Wojdasiewicz P , Gasik R , Szukiewicz D . Transforming growth factor Beta family: insight into the role of growth factors in regulation of fracture healing biology and potential clinical applications. Mediators Inflamm. 2015;2015:1‐17. 10.1155/2015/137823 PMC4325469 25709154

[35] Runyan CE , Schnaper HW , Poncelet AC . The role of internalization in transforming growth factor beta1‐induced Smad2 association with Smad anchor for receptor activation (SARA) and Smad2‐dependent signaling in human mesangial cells. J Biol Chem. 2005;280(9):8300‐8308. 15613484 10.1074/jbc.M407939200

[36] Gueorguieva I , Cleverly AL , Stauber A , et al. Defining a therapeutic window for the novel TGF‐beta inhibitor LY2157299 monohydrate based on a pharmacokinetic/pharmacodynamic model. Br J Clin Pharmacol. 2014;77(5):796‐807. 24868575 10.1111/bcp.12256 PMC4004400

[37] Al Shoyaib A , Archie SR , Karamyan VT . Intraperitoneal route of drug administration: should it Be used in experimental animal studies? Pharm Res. 2019;37(1):12. 31873819 10.1007/s11095-019-2745-x PMC7412579

[38] Hubert P , Roncarati P , Demoulin S , et al. Extracellular HMGB1 blockade inhibits tumor growth through profoundly remodeling immune microenvironment and enhances checkpoint inhibitor‐based immunotherapy. J Immunother Cancer. 2021;9(3):e001966. 33712445 10.1136/jitc-2020-001966 PMC7959241

[39] Chafe SC , McDonald PC , Saberi S , et al. Targeting hypoxia‐induced carbonic anhydrase IX enhances immune‐checkpoint blockade locally and systemically. Cancer Immunol Res. 2019;7(7):1064‐1078. 31088846 10.1158/2326-6066.CIR-18-0657

[40] de Graauw M , van Miltenburg MH , Schmidt MK , et al. Annexin A1 regulates TGF‐beta signaling and promotes metastasis formation of basal‐like breast cancer cells. Proc Natl Acad Sci USA. 2010;107(14):6340‐6345. 20308542 10.1073/pnas.0913360107 PMC2852023

[41] Schrors B , Boegel S , Albrecht C , et al. Multi‐omics characterization of the 4T1 murine mammary gland tumor model. Front Oncol. 2020;10:1195. 32793490 10.3389/fonc.2020.01195 PMC7390911

[42] Sanjabi S , Oh SA , Li MO . Regulation of the immune response by TGF‐beta: from conception to autoimmunity and infection. Cold Spring Harb Perspect Biol. 2017;9(6): a022236. 10.1101/cshperspect.a022236 PMC5453394 28108486

[43] Hua W , Ten Dijke P , Kostidis S , Giera M , Hornsveld M . TGFbeta‐induced metabolic reprogramming during epithelial‐to‐mesenchymal transition in cancer. Cell Mol Life Sci. 2020;77(11):2103‐2123. 31822964 10.1007/s00018-019-03398-6 PMC7256023

[44] Jin CH , Krishnaiah M , Sreenu D , et al. Discovery of N‐((4‐([1,2,4]triazolo[1,5‐a]pyridin‐6‐yl)‐5‐(6‐methylpyridin‐2‐yl)‐1H‐imidazol‐2‐yl)methyl)‐2‐fluoroaniline (EW‐7197): a highly potent, selective, and orally bioavailable inhibitor of TGF‐beta type I receptor kinase as cancer immunotherapeutic/antifibrotic agent. J Med Chem. 2014;57(10):4213‐4238. 24786585 10.1021/jm500115w

[45] Zhang H , Yao M , Morrison RA , Chong S . Commonly used surfactant, tween 80, improves absorption of P‐glycoprotein substrate, digoxin, in rats. Arch Pharm Res. 2003;26(9):768‐772. 14560928 10.1007/BF02976689

[46] Schwartzberg LS , Navari RM . Safety of polysorbate 80 in the oncology setting. Adv Ther. 2018;35(6):754‐767. 29796927 10.1007/s12325-018-0707-z PMC6015121

[47] Rouzier R , Perou CM , Symmans WF , et al. Breast cancer molecular subtypes respond differently to preoperative chemotherapy. Clin Cancer Res. 2005;11(16):5678‐5685. 16115903 10.1158/1078-0432.CCR-04-2421

[48] O'Meara TA , Tolaney SM . Tumor mutational burden as a predictor of immunotherapy response in breast cancer. Oncotarget. 2021;12(5):394‐400. 33747355 10.18632/oncotarget.27877 PMC7939529

[49] Driscoll CB , Schuelke MR , Kottke T , et al. APOBEC3B‐mediated corruption of the tumor cell immunopeptidome induces heteroclitic neoepitopes for cancer immunotherapy. Nat Commun. 2020;11(1):790. 32034147 10.1038/s41467-020-14568-7 PMC7005822

[50] Wang Y , Wu S , Zheng S , et al. APOBEC3G acts as a therapeutic target in mesenchymal gliomas by sensitizing cells to radiation‐induced cell death. Oncotarget. 2017;8(33):54285‐54296. 28903341 10.18632/oncotarget.17348 PMC5589580

[51] Liang G , Liu G , Kitamura K , et al. TGF‐β suppression of HBV RNA through AID‐dependent recruitment of an RNA exosome complex. PLoS Pathog. 2015;11(4):e1004780. 25836330 10.1371/journal.ppat.1004780 PMC4383551

[52] Dorman SN , Baranova K , Knoll JH , et al. Genomic signatures for paclitaxel and gemcitabine resistance in breast cancer derived by machine learning. Mol Oncol. 2016;10(1):85‐100. 26372358 10.1016/j.molonc.2015.07.006 PMC5528934

[53] Samstein RM , Lee CH , Shoushtari AN , et al. Tumor mutational load predicts survival after immunotherapy across multiple cancer types. Nat Genet. 2019;51(2):202‐206. 30643254 10.1038/s41588-018-0312-8 PMC6365097

[54] Schumacher TN , Schreiber RD . Neoantigens in cancer immunotherapy. Science. 2015;348(6230):69‐74. 25838375 10.1126/science.aaa4971

[55] Esebanmen GE , Langridge WHR . The role of TGF‐beta signaling in dendritic cell tolerance. Immunol Res. 2017;65(5):987‐994. 28845509 10.1007/s12026-017-8944-9

[56] Ganesh K , Massague J . TGF‐beta inhibition and immunotherapy: checkmate. Immunity. 2018;48(4):626‐628. 29669246 10.1016/j.immuni.2018.03.037 PMC6347120

[57] Hwang D , Vinod N , Skoczen SL , et al. Bioequivalence assessment of high‐capacity polymeric micelle nanoformulation of paclitaxel and Abraxane(R) in rodent and non‐human primate models using a stable isotope tracer assay. Biomaterials. 2021;278:121140. 34634661 10.1016/j.biomaterials.2021.121140 PMC10726948

