# Supplementary material for: Combination of polymeric micelle formulation of TGFβ receptor inhibitors and paclitaxel produces consistent response across different mouse models of Triple‐negative breast cancer
Source: Bioeng Transl Med. 2024 Jun 4;9(5):e10681. doi: 10.1002/btm2.10681 (PMC11561794; doi:10.1002/btm2.10681)
Supplement: Supplementary file 1 — Data S1. Supplementary Information. [file BTM2-9-e10681-s001.docx]

**Combination of Polymeric Micelle Formulation of TGFβ Receptor Inhibitors and Paclitaxel Produce Consistent Response Across Different Mouse Models of TNBC**

Natasha Vinod^1,2,#,+^, Duhyeong Hwang^1,3,#^, Sloane Christian Fussell^4,&^, Tyler Cannon Owens^1^, Olaoluwa Christopher Tofade^1^, Thad S. Benefield^5^, Sage Copling^1^, Jacob D. Ramsey^1^, Patrick D. Rädler^6,7^, Hannah M. Atkins^6,8,9,10^, Eric E. Livingston^11^, J. Ashley Ezzell^12^, Marina Sokolsky-Papkov^1^, Hong Yuan^11^, Charles M. Perou^6,7^ and Alexander V. Kabanov^1,^*

^1^Center for Nanotechnology in Drug Delivery and Division of Pharmacoengineering and Molecular Pharmaceutics, Eshelman School of Pharmacy, University of North Carolina at Chapel Hill, NC 27599, United States.

^2^Joint UNC/NC State Department of Biomedical Engineering, University of North Carolina, Chapel Hill, NC 27599, United States.

^3^College of Pharmacy, Keimyung University, Daegu 42601, Republic of Korea.

^4^Department of Biology, Department of Chemistry, University of North Carolina at Chapel Hill, Chapel Hill, NC, 27599, USA.

^5^Department of Radiology, University of North Carolina at Chapel Hill, Chapel Hill, NC 27514, United States.

^6^Lineberger Comprehensive Cancer Center, University of North Carolina, Chapel Hill, NC 27599, United States.

^7^Department of Genetics, University of North Carolina, Chapel Hill, NC 27514, United States.

^8^Pathology and Laboratory Medicine, School of Medicine, University of North Carolina, Chapel Hill, NC 27599, United States

^9^Department of Pathology and Laboratory Medicine, Division of Comparative Medicine, University of North Carolina, Chapel Hill, NC, 27599, USA

^10^Center for Human Health and the Environment, North Carolina State University, Raleigh, NC, 27695, USA

^11^Biomedical Research Imaging Center, Department of Radiology, and UNC Lineberger Comprehensive Cancer Center, University of North Carolina at Chapel Hill, Chapel Hill, NC 27599, United States.

^12^Histology Research Core, University of North Carolina, Chapel Hill, NC 27599, United States.

^#^Authors contributed equally to this study.

*Corresponding Author: Center for Nanotechnology in Drug Delivery, UNC Eshelman School of Pharmacy, University of North Carolina at Chapel Hill, 125 Mason Farm Road, Marsico Hall, Office #2012, Campus Box 7362, Chapel Hill, NC 27599-7362, USA, Tel: +1 (919) 537-3800. E-mail: [kabanov@email.unc.edu](mailto:kabanov@email.unc.edu)

^+^Present address: **Vinod, N,** National Cancer Institute, National Institutes of Health, Bethesda, MD 20892, United States.

^&^Present address: **Sloane Christian Fussell**, Vaccine Research Center, National Institute of Allergy and Infectious Disease, National Institutes of Health, Bethesda, MD 20892, United States.

**Power analysis of animal studies**

Figure 3

A factorial design with two factors at 6 and 7 levels has 42 cells (treatment combinations). A total of 126 subjects are required to provide 3 subjects per cell. The within-cell standard deviation is 138.000. This design achieves 83% power when an F test is used to test factor A (treatment) at a 5% significance level and the actual standard deviation among the appropriate means is 46.637 (an effect size of 0.338) and achieves 100% power when an F test is used to test factor B (time) at a 5% significance level and the actual standard deviation among the appropriate means is 112.609 (an effect size of 0.816).

Figure 4

A factorial design with two factors at 9 and 7 levels has 63 cells (treatment combinations). A total of 189 subjects are required to provide 3 subjects per cell. The within-cell standard deviation is 102.000. This design achieves 94% power when an F test is used to test factor A (treatment) at a 5% significance level and the actual standard deviation among the appropriate means is 35.428 (an effect size of 0.347) and achieves 100% power when an F test is used to test factor B (time) at a 5% significance level and the actual standard deviation among the appropriate means is 75.180 (an effect size of 0.737).

Figure 5

A factorial design with two factors at 9 and 7 levels has 63 cells (treatment combinations). A total of 252 subjects are required to provide 4 subjects per cell. The within-cell standard deviation is 185.000. This design achieves 98% power when an F test is used to test factor A (treatment) at a 5% significance level and the actual standard deviation among the appropriate means is 63.020 (an effect size of 0.341) and achieves 100% power when an F test is used to test factor B (time) at a 5% significance level and the actual standard deviation among the appropriate means is 147.533 (an effect size of 0.797).

Figure 6A

A factorial design with two factors at 6 and 9 levels has 54 cells (treatment combinations). A total of 216 subjects are required to provide 4 subjects per cell. The within-cell standard deviation is 219.000. This design achieves 100% power when an F test is used to test factor A (treatment) at a 5% significance level and the actual standard deviation among the appropriate means is 92.393 (an effect size of 0.422) and achieves 100% power when an F test is used to test factor B (time) at a 5% significance level and the actual standard deviation among the appropriate means is 146.755 (an effect size of 0.670).

Figure 6B

A factorial design with two factors at 6 and 7 levels has 42 cells (treatment combinations). A total of 168 subjects are required to provide 4 subjects per cell. The within-cell standard deviation is 136.000. This design achieves 100% power when an F test is used to test factor A (treatment) at a 5% significance level and the actual standard deviation among the appropriate means is 58.928 (an effect size of 0.433) and achieves 100% power when an F test is used to test factor B (time) at a 5% significance level and the actual standard deviation among the appropriate means is 87.962 (an effect size of 0.647).

**
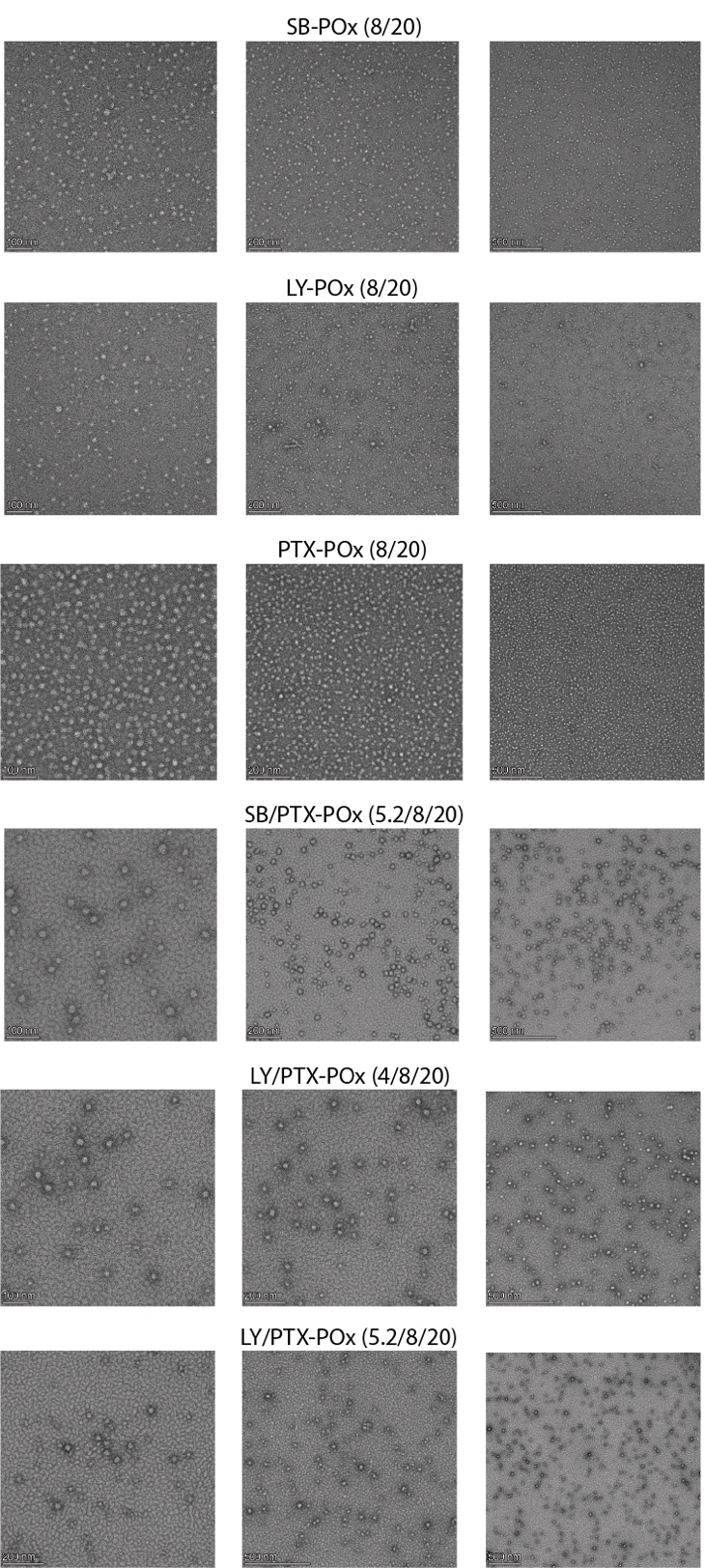
**

**Fig. S1 TEM images of the nanoassemblies formed in drug-loaded PMs of various compositions SB-POx (8/20); LY-POx (8/20); PTX-POx (8/20); SB/PTX-POx (5.2/8/20); LY/PTX-POx (4/8/20); LY/PTX-POx (5.2/8/20)**


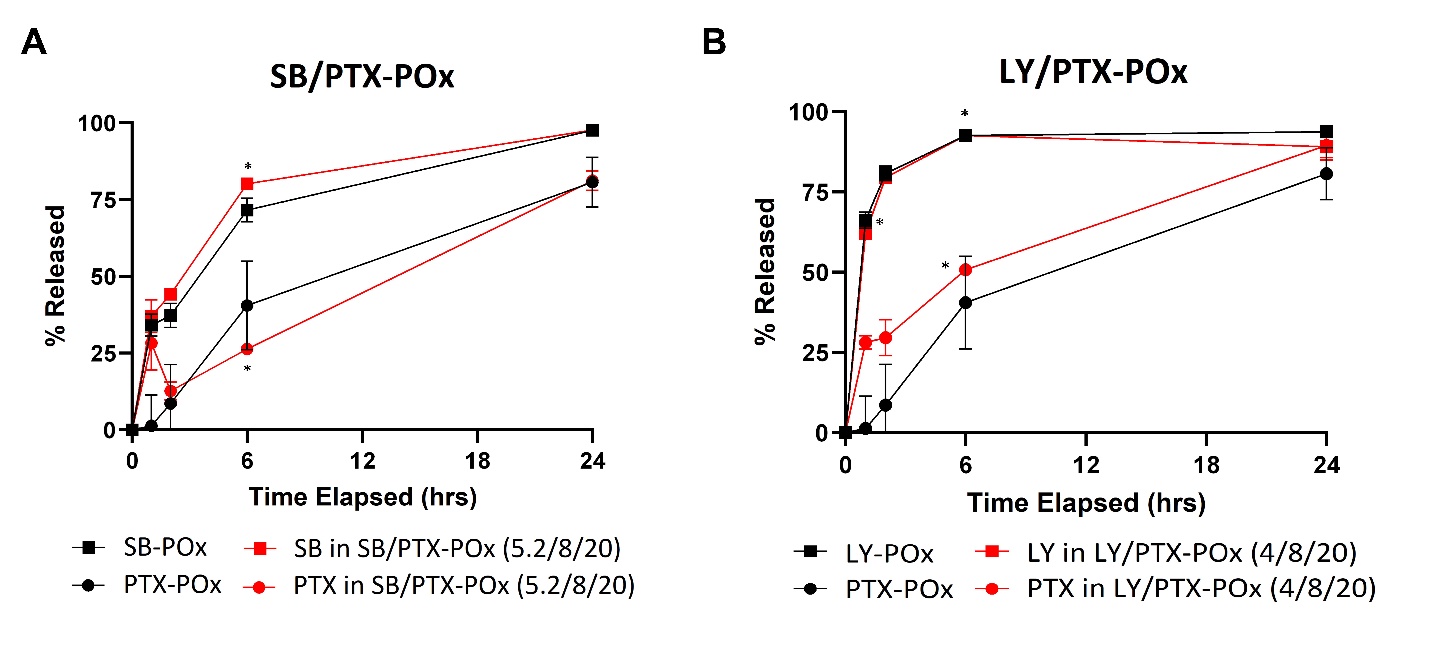
**Fig. S2 Drug release profiles of TGFβi or PTX from single and combination drug PM containing PTX and (A) SB or (B) LY**. The drug/polymer feed ratios were 5.2/8/20 **(A)** or 4/8/20 **(B)** for combination PM and 8/10 **(A, B)** for single drug PM. Data represent mean ± SD, (n = 3)**.** A common PTX-POx graph was used for both figures. *Datapoints with fewer than three replicates due to the datum being unusable because of a leak in the membrane of the dialysis device.


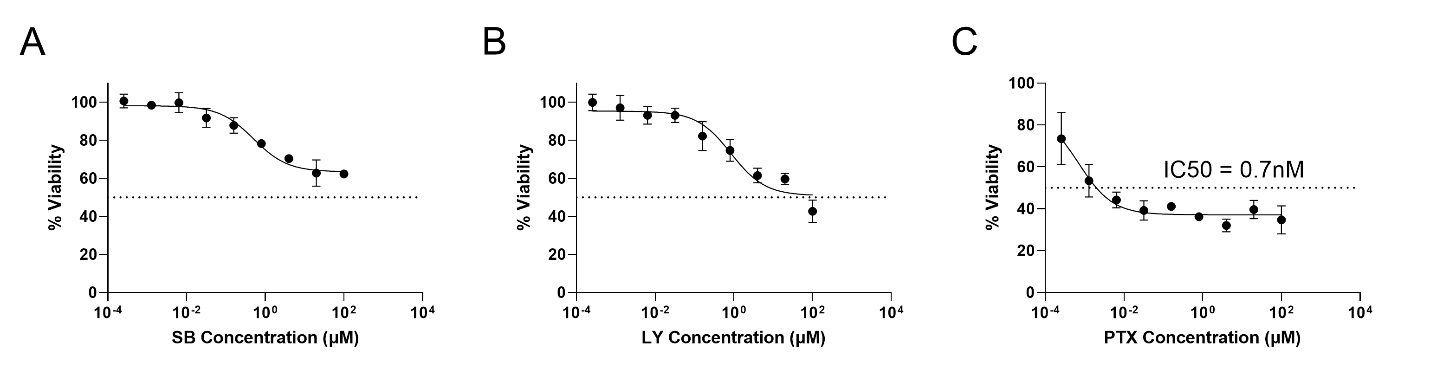
**Fig. S3 *In vitro* cytotoxicity of TGFβi and PTX in 4T1 cell line** Cell viability percent following 24h treatment with (**A**) SB-POx (**B**) LY-POx (**C**) PTX-POx.


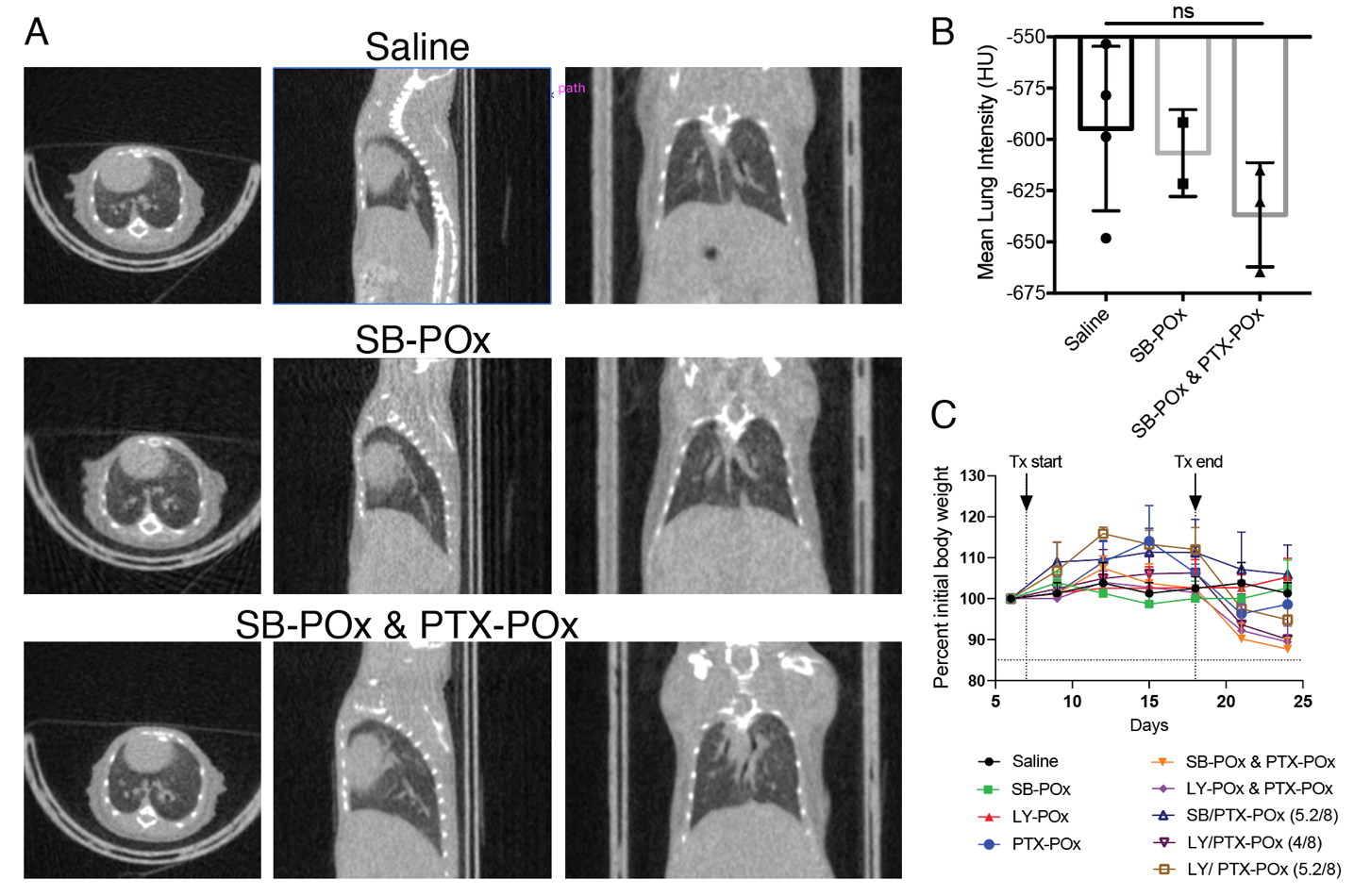


**Fig. S4** **Efficacy of intraperitoneally delivered TGFβi against primary and metastatic tumors** (**A**) Percent tumor growth inhibition corresponding to tumor growth curves in 3C and 3D (**B**) Microtomography quantification of mean lung intensity expressed in the Hounsfield units. Statistical comparisons were computed by one-way ANOVA with Tukey’s multiple comparisons test. (**C**) Body weight changes (percent of initial) in mice treated with TGFβi PM and/or PTX PM.

**Table S1**: Experimental conditions for the preparation of POx formulations of TGFβi and PTX

| Formulation | Stock concentration of drugs in ethanol (mg/ml) | Evaporation temperature (^0^C) | Hydration temperature (^0^C) | Hydration time  (min) |
| --- | --- | --- | --- | --- |
| SB-POx | 10 | 50 | RT | 20 |
| LY-POx | 5 | 65 | Intermittent heating at 65 | 5 |
| PTX-POx | 10 | 45 | 65 | 20 |
| SB/PTX-POx (5.2/8) |  | 50 | Intermittent heating at 65 | 5 |
| LY/PTX-POx (4/8) |  | 60 | Intermittent heating at 60 | 5 |
| LY/PTX-POx (5.2/8) |  | 65 | Intermittent heating at 65 | 5 |

**Table S2**: Details of antibodies used in Western blot and flow cytometry experiments

| Antibody | Manufacturer | Clone | Host | Antibody Dilution | Lysate concentration |
| --- | --- | --- | --- | --- | --- |
| β-actin | Cell Signaling Technology | 13E5 | Rabbit | 1:50 | 0.4 mg/mL |
| p-Smad2/3(pThr^8^) | Sigma-Aldrich | Polyclonal | Rabbit | 1:100 | 0.4 mg/mL |
| PE anti-p-Smad2 (pS465/pS467) /Smad3(pS423/pS425) | BD Biosciences | 072-670 | Human | 1:20 | - |

**Table S3** 4T1 model. Statistical analyses of data corresponding to Figure 3A. By two-way ANOVA with Tukey’s test for multiple comparisons. Statistical difference: **** (p < 0.0001). (Graphpad Prism, version 8.4.3).

|  | PTX-POx (8/20) | SB-POx (8/20)  daily | SB-POx (8/20)  eod | SB-POx (8/20) daily + PTX-POx (8/20) | SB-POx (8/20) eod + PTX-POx (8/20) |
| --- | --- | --- | --- | --- | --- |
| Saline | **** | ns | ns | **** | **** |
| PTX-POx (8/20) |  | **** | **** | ns | ns |
| SB-POx (8/20) daily |  |  | ns | **** | **** |
| SB-POx (8/20) eod |  |  |  | **** | **** |
| SB-POx (8/20) daily  & PTX-POx (8/20) |  |  |  |  | ns |

**Table S4** Statistical analyses of data corresponding to Figure 3B. By one-way ANOVA with Tukey’s test for multiple comparisons. Statistical difference: **** (p < 0.0001). (Graphpad Prism, version 9).

|  | SB-POx eod | SB-POx daily & PTX-POx | SB-POx eod & PTX-POx | PTX-POx |
| --- | --- | --- | --- | --- |
| SB-POx daily | ns | * | ** | * |
| SB-POx eod |  | * | ** | * |
| SB-POx daily & PTX-POx |  |  | ns | ns |
| SB-POx eod & PTX-POx |  |  |  | ns |

**Table S5** 4T1 model. Statistical analyses of data corresponding to Figures 4A and 4B. By two-way ANOVA with Tukey’s test for multiple comparisons. Statistical difference: * (p < 0.05), ** (p < 0.01), *** (p < 0.001), and **** (p < 0.0001). (Graphpad Prism, version 8.4.3).

|  | PTX PM | SB PM | LY PM | SB PM + PTX PM | LY PM + PTX PM | SB/PTX (5.2/8) PM | LY/PTX (4/8) PM | LY/PTX (5.2/8) PM |
| --- | --- | --- | --- | --- | --- | --- | --- | --- |
| Saline | **** | ** | ns | **** | **** | ns | **** | **** |
| PTX-POx |  | ns | ns | ns | ns | ns | ns | ns |
| SB-POx |  |  | ns | ns | * | ns | ns | ns |
| LY-POx |  |  |  | **** | **** | ns | ** | ns |
| SB-POx & PTX-POx |  |  |  |  | ns | *** | ns | ns |
| LY-POx & PTX PM |  |  |  |  |  | **** | ns | * |
| SB/PTX (5.2/8) PM |  |  |  |  |  |  | * | ns |
| LY/PTX (4/8) PM |  |  |  |  |  |  |  | ns |


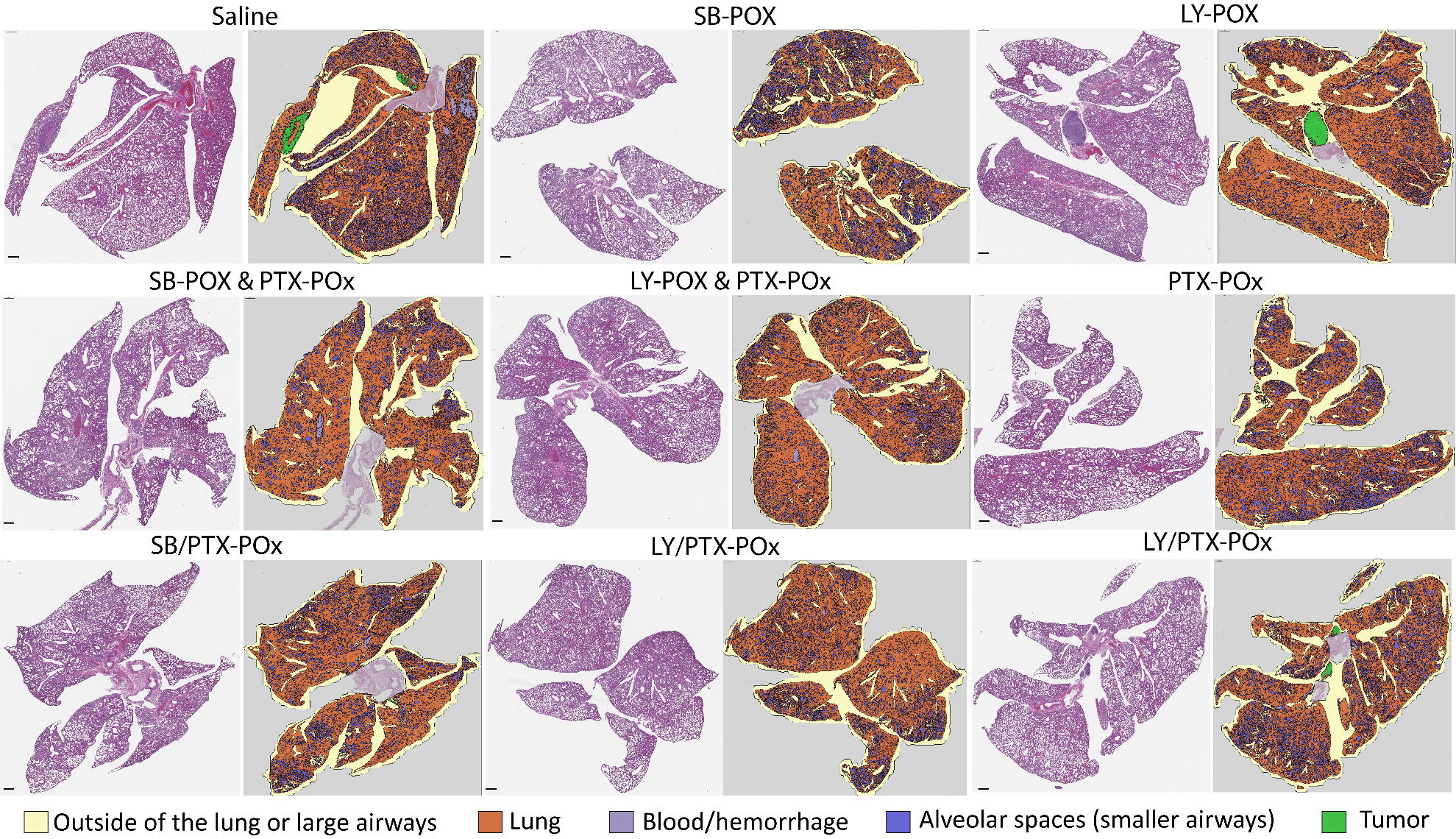


**Fig. S5 Micrometastases in the lungs of mice treated with PTX-POx and/or intraperitoneal TGFβi-Px.** Representative images of the H&E staining of mouse lungs (left) corresponding to Figure 4D with the image analysis software overlay delineating the tumor margins in green (right).

**Table S6** 4T1 model. Statistical analyses of data corresponding to Figure 5A. By two-way ANOVA with Tukey’s test for multiple comparisons. Statistical difference: * (p < 0.05), ** (p < 0.01), *** (p < 0.001), and **** (p < 0.0001). (Graphpad Prism, version 8.4.3).

|  | PTX-POx | o.g. SB- POx | o.g. LY-POx | o.g. SB-POx  & PTX-POx | o.g. LY-POx  & PTX-POx | o.g. SB | o.g LY | o.g. SB  & PTX-POx | o.g. LY  & PTX-POx |
| --- | --- | --- | --- | --- | --- | --- | --- | --- | --- |
| Saline | ** | ** | ns | **** | ns | ns | ns | **** | **** |
| PTX PM |  | **** | ** | ns | ns | *** | * | ns | ns |
| o.g. SB PM |  |  | ** | **** | **** | * | *** | **** | **** |
| o.g. LY PM |  |  |  | **** | ns | ns | ns | **** | **** |
| o.g. SB PM & PTX PM |  |  |  |  | ** | **** | **** | ns | ns |
| o.g. LY PM & PTX PM |  |  |  |  |  | * | ns | *** | ns |
| o.g. SB |  |  |  |  |  |  | ns | **** | **** |
| o.g. LY |  |  |  |  |  |  |  | **** | *** |
| o.g. SB & PTX PM |  |  |  |  |  |  |  |  | ns |

**Table S7** Statistical analyses of data corresponding to Figure 5B. By one-way ANOVA with Tukey’s test for multiple comparisons. Statistical difference: **** (p < 0.0001). (Graphpad Prism, version 9).

|  | o.g. LY-POx | o.g. SB | o.g. LY | PTX-POx | o.g. SB-POx & PTX-POx | o.g. LY-POx & PTX-POx | o.g. SB & PTX-POx | o.g. LY & PTX-POx |
| --- | --- | --- | --- | --- | --- | --- | --- | --- |
| o.g. SB-POx | * | ns | ns | **** | **** | **** | **** | **** |
| o.g. LY-POx |  | ns | ns | ns | ** | ns | **** | * |
| o.g. SB |  |  | ns | ** | *** | * | **** | ** |
| o.g. LY |  |  |  | * | *** | ns | **** | ** |
| PTX-POx |  |  |  |  | ns | ns | ns | ns |
| o.g. SB-POx & PTX-POx |  |  |  |  |  | ns | ns | ns |
| o.g. LY-POx & PTX-POx |  |  |  |  |  |  | ns | ns |
| o.g. SB & PTX-POx |  |  |  |  |  |  |  | ns |


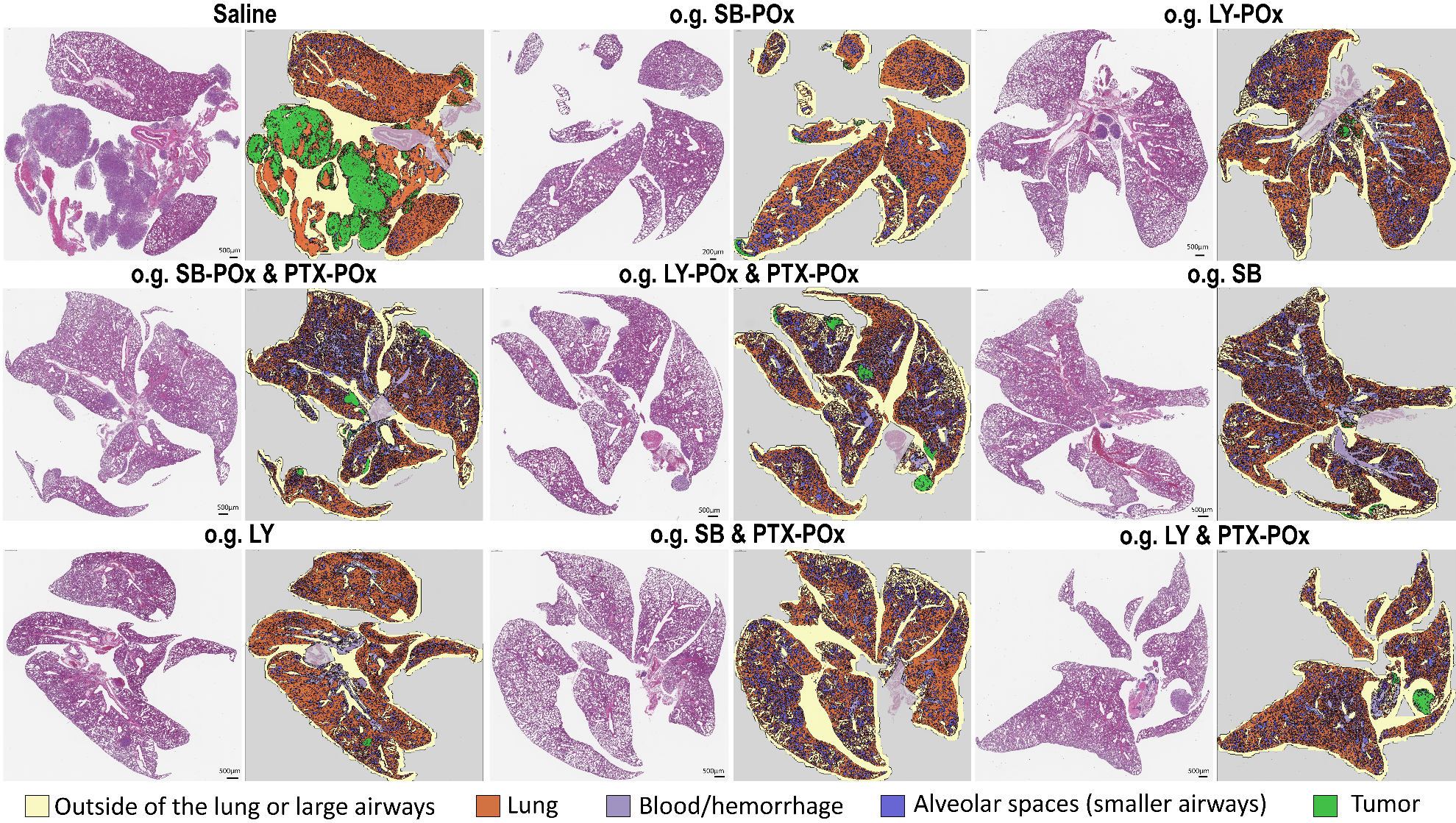


**Fig. S6 Micrometastases in the lungs of mice treated with PTX-POx and/or oral TGFβi.** Representative images of the H&E staining of mouse lungs (left) corresponding to Figure 5C with the image analysis software overlay delineating the tumor margins in green (right).

**Table S8** T11-Apobec model. Statistical analyses of data corresponding to Figure 6A. By two-way ANOVA with Tukey’s test for multiple comparisons. Statistical difference: * (p < 0.05), ** (p < 0.01), *** (p < 0.001), and **** (p < 0.0001). (Graphpad Prism, version 8.4.3).

|  | PTX PM | SB PM | LY PM | SB PM + PTX PM | LY PM + PTX PM |
| --- | --- | --- | --- | --- | --- |
| Saline | **** | **** | **** | **** | **** |
| PTX PM |  | ns | ns | * | *** |
| SB PM |  |  | ns | ns | * |
| LY PM |  |  |  | ns | ns |
| SB PM + PTX PM |  |  |  |  | ns |

**Table S9** T11-UV model. Statistical analyses of data corresponding to Figure 6B. By two-way ANOVA with Tukey’s test for multiple comparisons. Statistical difference: * (p < 0.05), ** (p < 0.01), *** (p < 0.001), and **** (p < 0.0001). (Graphpad Prism, version 8.4.3).

|  | PTX PM | SB PM | LY PM | SB PM + PTX PM | LY PM + PTX PM |
| --- | --- | --- | --- | --- | --- |
| Saline | **** | ns | ** | **** | **** |
| PTX PM |  | **** | *** | ns | ns |
| SB PM |  |  | ns | * | *** |
| LY PM |  |  |  | ns | ** |
| SB PM + PTX PM |  |  |  |  | ns |

A


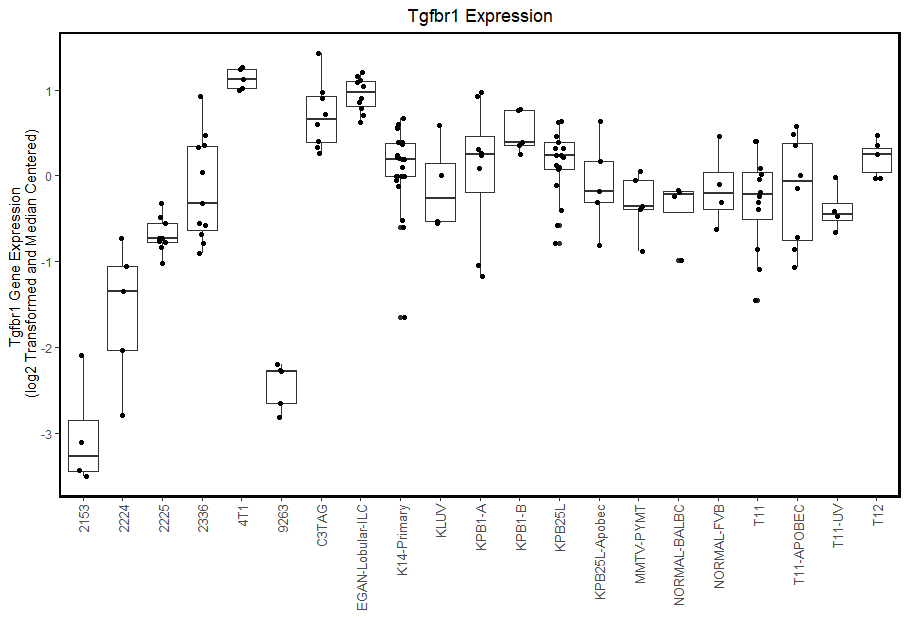


B


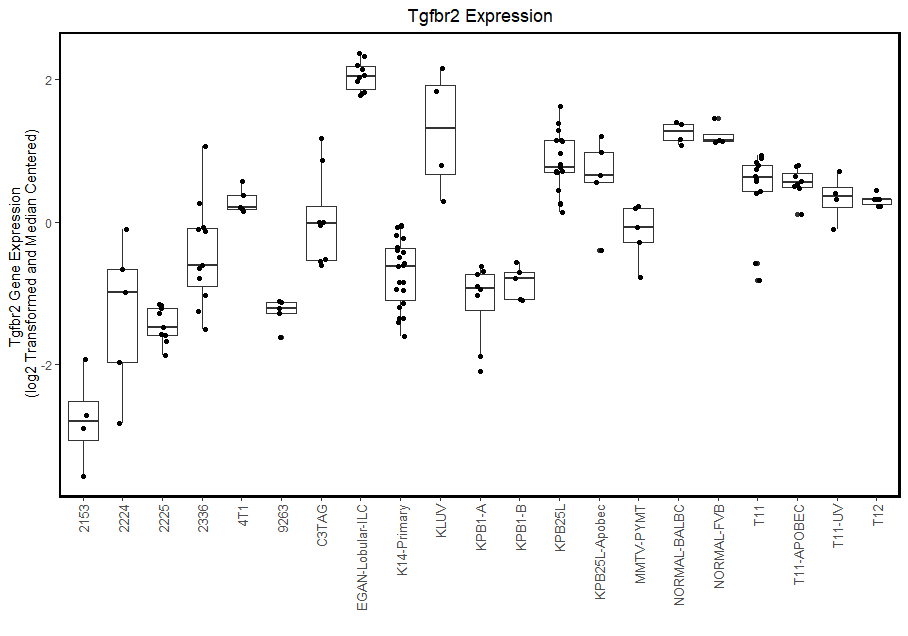


C


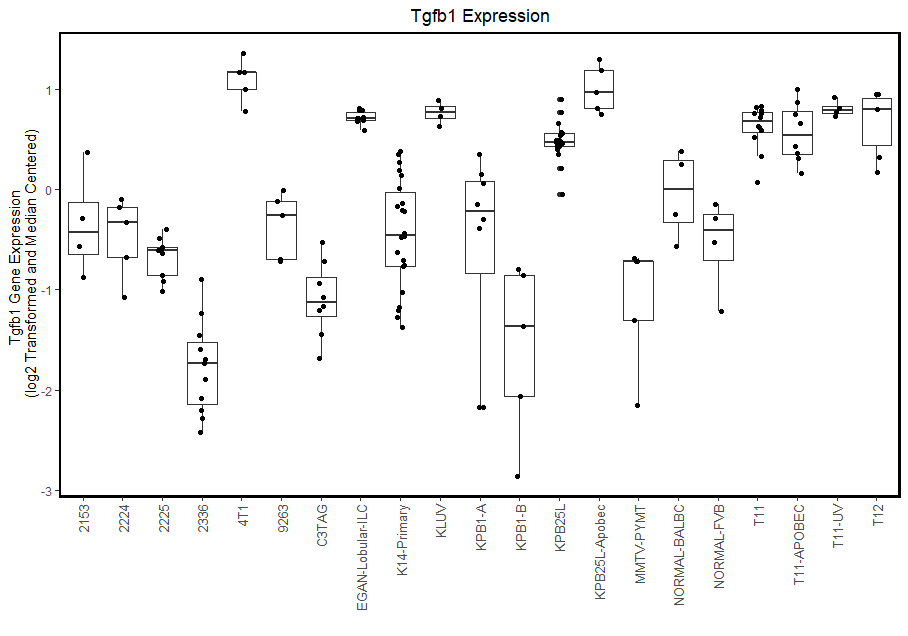


D


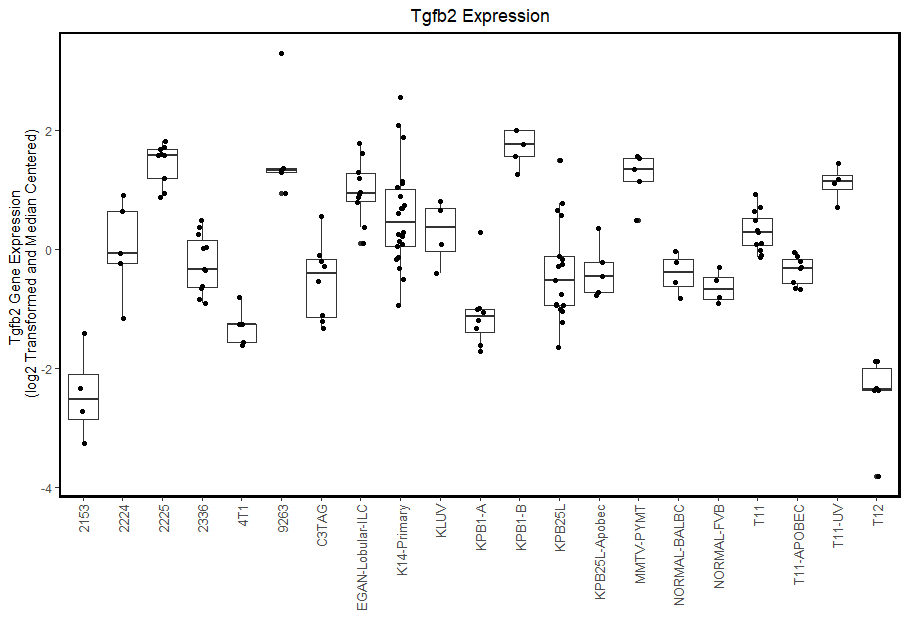


E


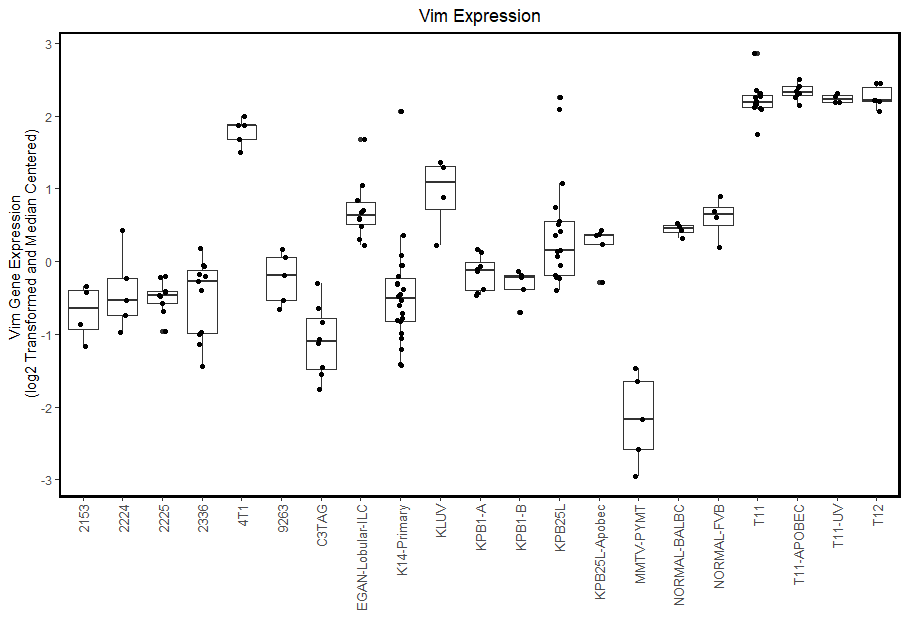


F


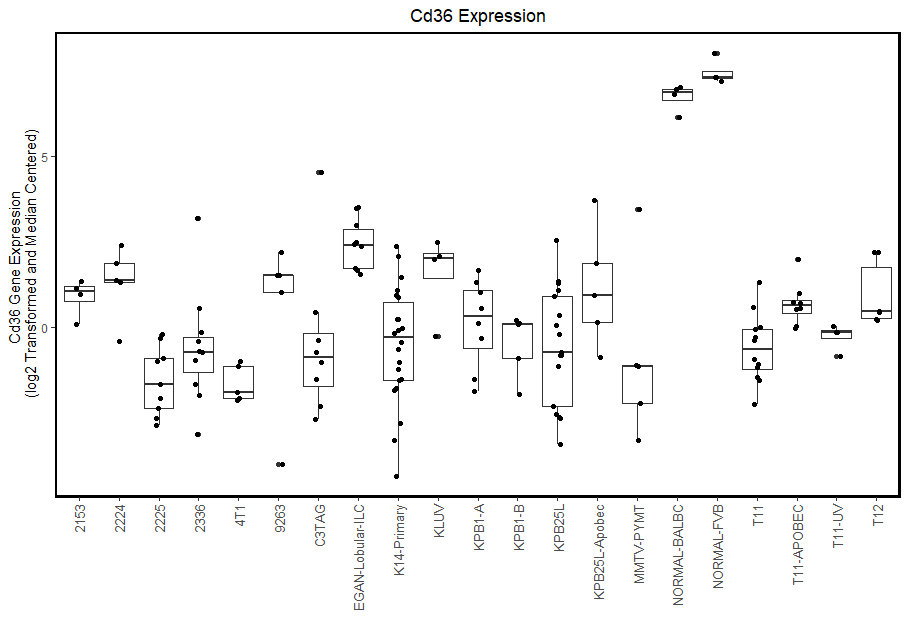


G


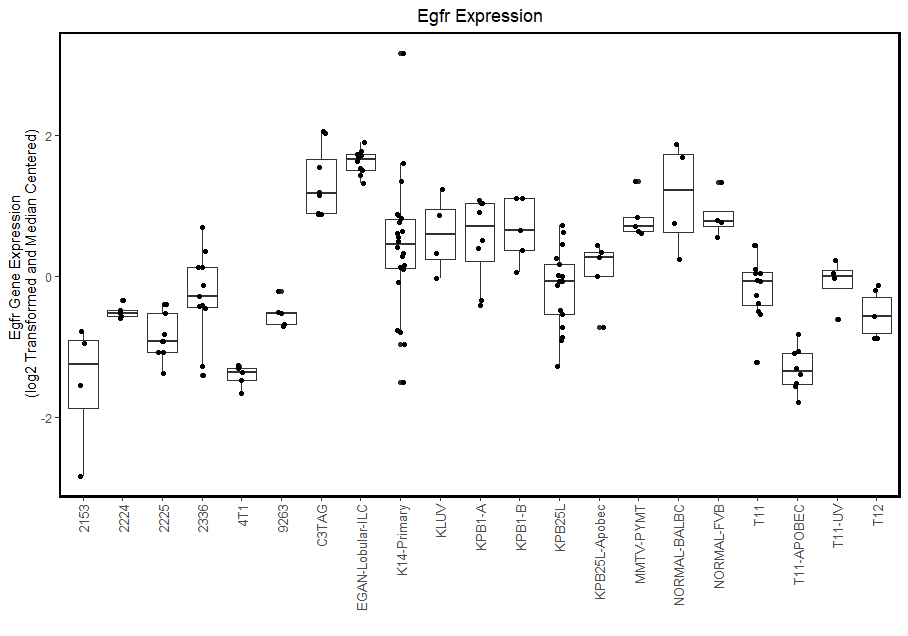


H


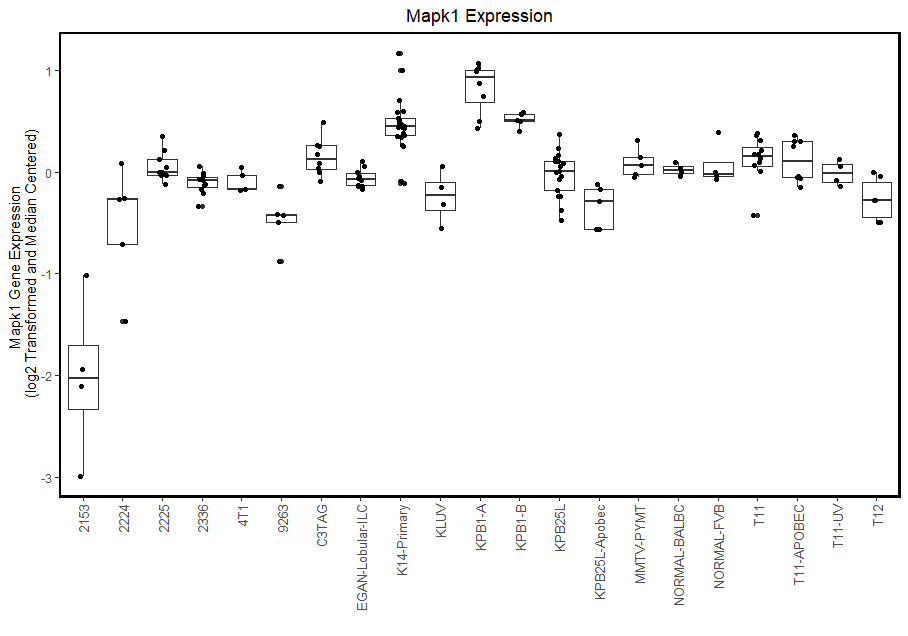


I


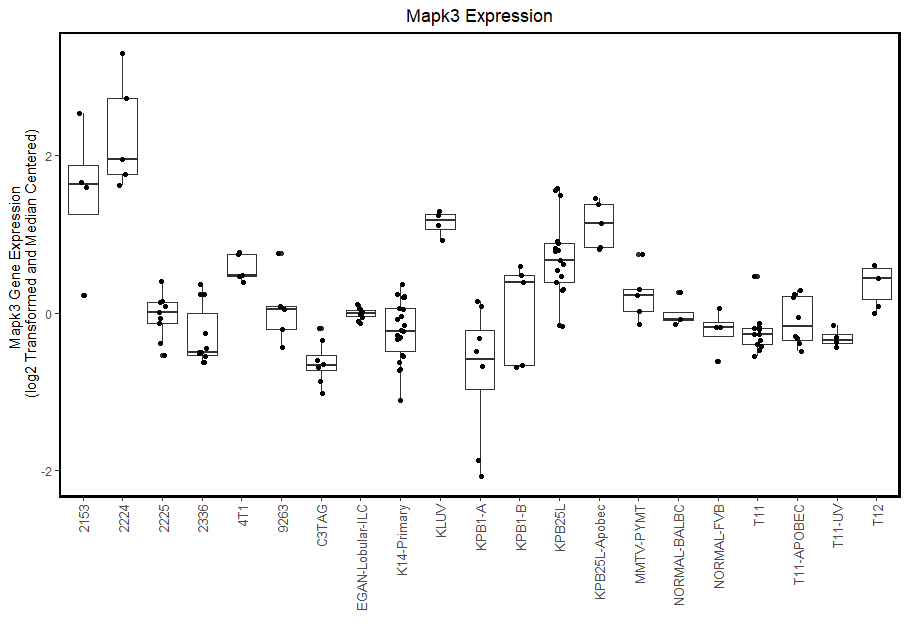


J


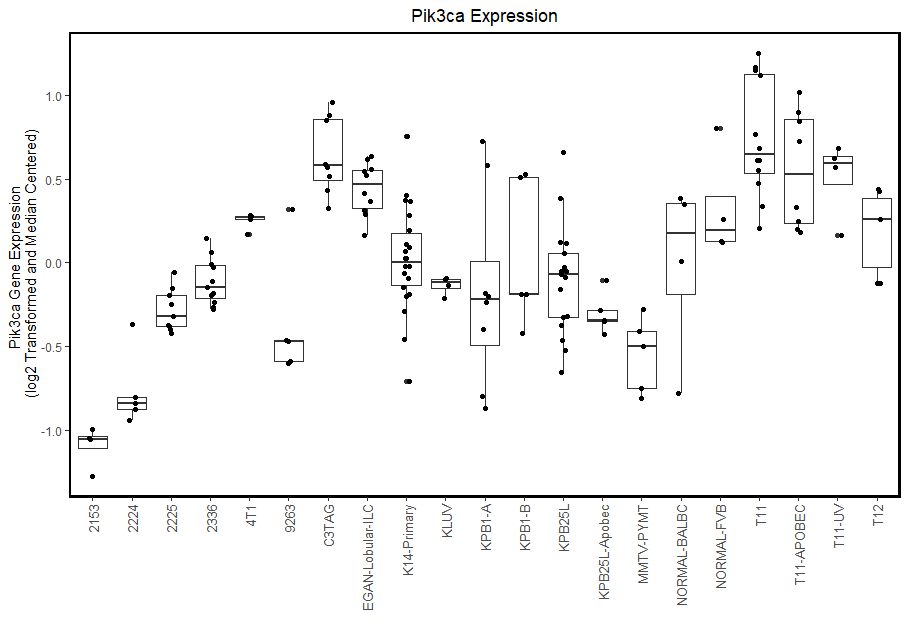


K


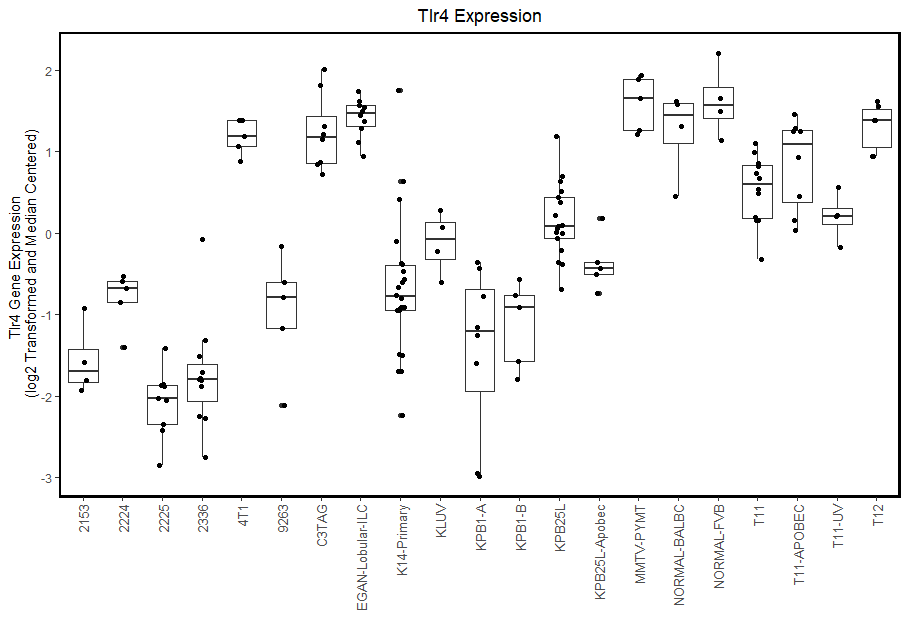


L


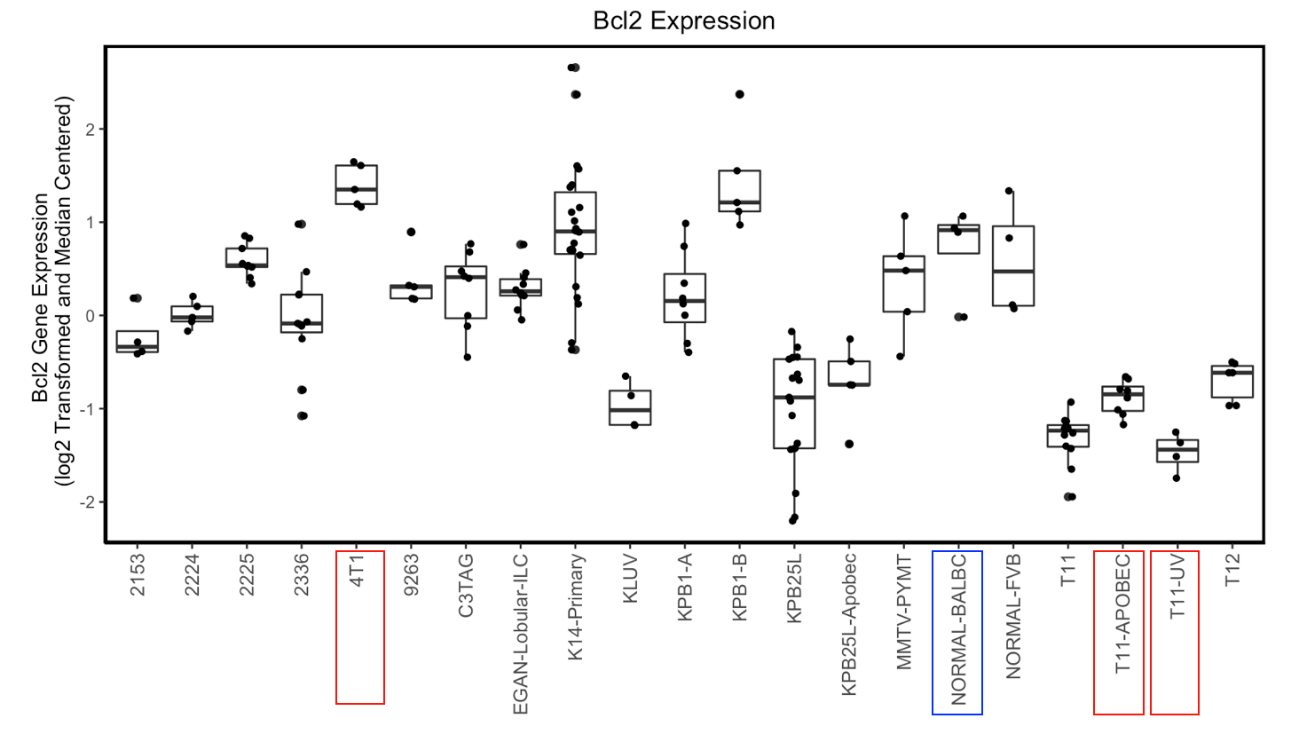


**Fig. S7.** Transcriptome evaluation (RNA-seq) of 19 TNBC models and 2 normal mammary gland gene expression profiles. A – transforming growth factor beta receptor 1 (Tgfbr1), B – transforming growth factor beta receptor 2 (Tgfbr2), C – transforming growth factor beta 1 (Tgfb1), D – transforming growth factor beta 2 (Tgfbr2), E – vimentin (Vim), F – CD36, G – Epidermal growth factor receptor (Egfr), H – Mitogen-Activated Protein Kinase 1 (Mapk1), I – Mitogen-Activated Protein Kinase 3 (Mapk3), J – Phosphatidylinositol-4,5-Bisphosphate 3-Kinase Catalytic Subunit Alpha (Pik3ca), K – toll-like receptor 4 (Tlr4), L – B-cell lymphoma 2 (Bcl2). The transcriptome sequencing of the mouse tumors and mammary glands was carried out as previously described.^1^

**Reference**

1. Hollern DP, Xu N, Thennavan A, et al. B Cells and T Follicular Helper Cells Mediate Response to Checkpoint Inhibitors in High Mutation Burden Mouse Models of Breast Cancer. *Cell*. Nov 14 2019;179(5):1191-1206 e21. doi:10.1016/j.cell.2019.10.028
